# Shifts in metabolic hydrogen sinks in the methanogenesis-inhibited ruminal fermentation: a meta-analysis

**DOI:** 10.3389/fmicb.2015.00037

**Published:** 2015-02-04

**Authors:** Emilio M. Ungerfeld

**Affiliations:** Instituto de Investigaciones Agropecuarias (INIA) CarillancaTemuco, Chile

**Keywords:** methanogenesis inhibition, rumen, fermentation, metabolic hydrogen, meta-analysis, volatile fatty acids

## Abstract

Maximizing the flow of metabolic hydrogen ([H]) in the rumen away from CH_4_ and toward volatile fatty acids (VFA) would increase the efficiency of ruminant production and decrease its environmental impact. The objectives of this meta-analysis were: (i) To quantify shifts in metabolic hydrogen sinks when inhibiting ruminal methanogenesis *in vitro*; and (ii) To understand the variation in shifts of metabolic hydrogen sinks among experiments and between batch and continuous cultures systems when methanogenesis is inhibited. Batch (28 experiments, *N* = 193) and continuous (16 experiments, *N* = 79) culture databases of experiments with at least 50% inhibition in CH_4_ production were compiled. Inhibiting methanogenesis generally resulted in less fermentation and digestion in most batch culture, but not in most continuous culture, experiments. Inhibiting CH_4_ production in batch cultures resulted in redirection of metabolic hydrogen toward propionate and H_2_ but not butyrate. In continuous cultures, there was no overall metabolic hydrogen redirection toward propionate or butyrate, and H_2_ as a proportion of metabolic hydrogen spared from CH_4_ production was numerically smaller compared to batch cultures. Dihydrogen accumulation was affected by type of substrate and methanogenesis inhibitor, with highly fermentable substrates resulting in greater redirection of metabolic hydrogen toward H_2_ when inhibiting methanogenesis, and some oils causing small or no H_2_ accumulation. In both batch and continuous culture, there was a decrease in metabolic hydrogen recovered as the sum of propionate, butyrate, CH_4_ and H_2_ when inhibiting methanogenesis, and it is speculated that as CH_4_ production decreases metabolic hydrogen could be increasingly incorporated into formate, microbial biomass, and perhaps, reductive acetogenesis in continuous cultures. Energetic benefits of inhibiting methanogenesis depended on the inhibitor and its concentration and on the *in vitro* system.

## Introduction

Despite its importance in transforming fibrous plant biomass non-edible by humans into useful products such as meat and milk, ruminant production contributes with between 7 and 18% of total anthropogenic greenhouse gas emissions, with CH_4_ release from ruminal fermentation being a principal contributor (Martin et al., 2010; Hristov et al., 2013). The release of CH_4_ produced in the rumen to the atmosphere is also energetically inefficient to animal production, as it accounts for between 2 and 12% of ruminant gross energy intake (Johnson and Johnson, 1995).

Glucose is mainly metabolized through glycolysis to pyruvate in the rumen (Russell and Wallace, 1997). Glycolysis, and pyruvate oxidative decarboxylation to acetyl-CoA, which is the first step in acetate and butyrate formation, both result in the release of metabolic hydrogen ([H]). Reduced co-factors must then be re-oxidized for fermentation to continue. In the typical ruminal fermentation, methanogenesis is the main route of co-factor re-oxidation, with [H] transferred from the fermentative microbiota of bacteria, protozoa and fungi to methanogenic Archaea mainly as H_2_ (Wolin et al., 1997). However, the production of propionate, a useful fermentation product which is the main glucose precursor for ruminants, competes with CH_4_ production for [H] (Janssen, 2010) and H_2_ (Henderson, 1980). Also, some [H] is incorporated into butyrate production from pyruvate (Miller and Jenesel, 1979); although butyrate production from hexoses results in net release of [H], less [H] per mol of glucose is released from butyrate formation compared to acetate (Janssen, 2010).

Because of the negative consequences to the environment and the energetic inefficiency that it represents, much research effort has been directed toward the inhibition of ruminal methanogenesis. Methane production can be strongly decreased using halogenated CH_4_ analogs and other chemicals, nitrate, oils, antiprotozoal agents or some plant extracts. Although there has been progress at decreasing CH_4_ production *in vitro* and *in vivo*, a problem encountered is the incomplete incorporation of [H] spared from methanogenesis into fermentation products nutritionally useful to the host animal. Although some [H] spared from CH_4_ production is redirected to propionate, part is directed to atypical [H] sinks like H_2_ (Kung et al., 2003; Mitsumori et al., 2012), and sometimes formate and ethanol (Ungerfeld et al., 2003). Accumulation of H_2_ is energetically inefficient and inhibits re-oxidation of co-factors and hence fermentation (Wolin et al., 1997). It would be important to incorporate more [H] spared from methanogenesis into propionate and butyrate production.

The profile of VFA and gases in the normal, non-inhibited ruminal fermentation has been modeled based on thermodynamics and kinetics (Kohn and Boston, 2000; Offner and Sauvant, 2006). Mechanistic explanations have been proposed for the typical range of VFA proportions (Ungerfeld and Kohn, 2006) and the acetate to propionate shift that occurs when ruminal methanogens are inhibited (Janssen, 2010). However, even though the general response in propionate and H_2_ to methanogenesis inhibition is well described, shifts in all the main [H] sinks from numerous experiments have not been quantitatively summarized. Furthermore, there is variation among *in vitro* systems and experiments in fermentation shifts when methanogenesis is inhibited, as the present analysis will show. Understanding this variation and identifying the underlying factors that can explain it may allow better manipulation of fermentation to direct [H] toward the most beneficial sinks.

To my knowledge, there have not been *in vivo* studies with simultaneous measurement of actual production of VFA (rather than concentration) and gases. On the other hand, there is a considerable amount of published *in vitro* results on the effects of inhibiting ruminal methanogenesis on net VFA and gases production. A meta-analytical approach can be used to quantitatively summarize results from the existing studies. Meta-analyses can be conducted with the objective of augmenting power in hypothesis testing, modeling responses of studies conducted under dissimilar conditions, establishing new research hypotheses based on aggregated results from many studies, or parameterizing models (Sauvant et al., 2008). Meta-analyses allow integration of studies conducted under disparate conditions, which can allow the emergence of new knowledge or hypotheses not provided by the individual experiments. Rather than establishing cause-effect relationships, meta-analyses can discover associations useful to identify new variables for further research. The objectives of the present meta-analysis are to: (i) Quantitatively summarize the effects of methanogenesis inhibition on [H] sinks in ruminal batch and continuous cultures; and (ii) Understand the underlying variation among *in vitro* systems and experiments in the shifts of [H] to different sinks. Possible implications of the findings from this analysis of *in vitro* experiments to the *in vivo* situation are discussed.

## Methods

### Databases

Experiments of inhibition of ruminal methanogenesis in batch and continuous cultures were compiled. In order to be included in this meta-analysis, experiments had to meet all of the following criteria:

- Production of CH_4_, H_2_ accumulation, and net production of individual VFA was provided or could be calculated;
- Initial headspace was H_2_-free and formate salts or formic acid were not used as substrate;
- The experiment included a methanogenesis-uninhibited control treatment;
- At least one treatment or level within a treatment resulted in a 50% or greater decrease in CH_4_ production relative to the control. This ensured sufficient variation in CH_4_ production in each individual experiment so as to adequately test the hypotheses that variation in the responses was related to methanogenesis inhibition;
- Experiments with very atypical VFA molar percentages in control treatments (Marty and Demeyer, 1973; Hino and Russell, 1985) were not included;
- Treatments within experiments consisting of combinations of methanogenesis inhibitors and fermentation intermediates or their isomers or analogs (malate, fumarate, crotonate, butynoic acid or 3-butenoic acid) were not included, in order to avoid confounding effects of added fermentation intermediates on VFA production unrelated to methanogenesis inhibition;
- In the batch culture study by Nollet et al. (1997), treatments with added reductive acetogen *Peptostreptococcus productus* were not included, as an unknown part of acetate produced when inhibiting methanogenesis could potentially be originated by reductive acetogenesis and hence would not be associated with production of [H].

Incubations including different treatments that were run simultaneously with the same uninhibited control were considered to be one experiment. The batch culture database comprised a total of 193 treatment means from 28 experiments in 14 peer-reviewed published studies (Table S1). The continuous cultures database comprised a total of 79 treatment means from 16 experiments in 13 peer-reviewed published studies (Table S2).

### Calculations

Total production of reducing equivalent pairs (*[2H]_produced_*) was calculated from the stoichiometry of reducing equivalent-pairs released in acetate (*Ac*), propionate (*Pr*) and butyrate (*But*) production (Marty and Demeyer, 1973) as:

(1) $${\left[2H\right]}_{produced}=2Ac+Pr+4\text{ But}$$

Production of valerate and caproate was not considered because some experiments reported only the three main VFA acetate, propionate and butyrate.

Total incorporation of reducing equivalent-pairs into the main fermentation products (*[2H]_incorporated_*) was calculated from the stoichiometry of reducing equivalent-pairs incorporated into propionate, butyrate, CH_4_ and H_2_(Marty and Demeyer, 1973):

(2) $${\left[2H\right]}_{incorporated}=2Pr+2But+4{\text{CH}}_{\text{4}}+{\text{H}}_{2}$$

Metabolic hydrogen recovery (*[2H]_recovery_*) was calculated as the ratio between total *[2H]_incorporated_* and *[2H]_produced_*, expressed as percentage (Marty and Demeyer, 1973):

(3) $${\left[2H\right]}_{recovery}={\left[2H\right]}_{incorporated}\times 100/{\left[2H\right]}_{produced}$$

Organic matter fermentation on a total fermented hexoses-equivalent basis (*FH*) was estimated according to Marty and Demeyer (1973) as:

(4) $$FH=\frac{1}{2}Ac+\frac{1}{2}Pr+But$$

All variables in the equations above are expressed in μmol (batch culture) or mmol/d (continuous culture).

Heat of combustion of glucose, acetic acid, propionic acid, butyric acid, CH_4_ and H_2_ were obtained from Domalski (1972) and Kohn and Boston (2000), and used to calculated heat of combustion output in total VFA (Δ*H_VFA_*) and in gases (CH_4_ + H_2_, Δ*H_gases_*).

### Regressions

The incorporation of [H] into propionate (*[2H]_Pr_*), butyrate (*[2H]_But_*) and H_2_ (*[2H]_H2_*), *[2H]_recovery_, FH*, Δ*H_VFA_* and Δ*H_gases_* were regressed separately against the incorporation of [H] into CH_4_ (*[2H]_CH4_*), for batch and continuous cultures as:

(5) $$\begin{array}{l} response_{ij}\;=\text{intercept}+exp_{i}+{\text{B}}_{1}{\left[2H\right]}_{CH4j}+{\text{B}}_{2}{\left[2H\right]}_{CH4j}^{2}\\ \;+\;{\text{b}}_{i}{\left[2H\right]}_{CH4ij}+residual_{ij} \end{array}$$

where *response_ij_* is *[2H]_Pr_, [2H]_But_, [2H]_H2_, [2H]_recovery_, FH*, Δ*H_VFA_* or Δ*H_gases_, exp_i_* is the fixed effect of the experiment i, B_1_ and B_2_ are fixed linear and quadratic regression coefficients of *[2H]_CH4_*, respectively, and b*_i_* is the fixed effect of experiment i on the linear *[2H]_CH4_* coefficient (i.e., interaction between the experiment effect and methanogenesis inhibition), with *residual_ij_* assumed to be independent and normally distributed. Fixed, rather than random, effect of the experiment, was used because the objective of the analysis was not to parameterize models to predict future *in vitro* results but to use the existing information to understand how methanogenesis inhibition affects the flows of [H] in ruminal fermentation. Regression coefficient estimates were compared to zero with a Student's test. Significance was declared at *p* < 0.05 and tendencies at 0.05 ≤ *p* ≤ 0.15. Non-significant interactions and quadratic effects (*p* > 0.15) were removed and the reduced model re-run.

In meta-analysis, weighting treatment means by the reciprocal of their standard errors (1/SEM) scaled to one is recommended to obtain maximum likelihood estimates while maintaining the original scale of the data (Sauvant et al., 2008). Unfortunately, SEM were not always available for the production of individual VFA, because for many experiments production of individual VFA had to be calculated as the product of total production of VFA by their molar proportions, hence the SEM for the resulting variable is not calculable from the published SEM. Total VFA concentration 1/SEM was instead initially chosen as weighting variable for all regressions (1/SEM of CH_4_ production was not chosen because CH_4_ production was small or non-detectable in some treatments, which associated with null or very small reported SEM and thus very large 1/SEM). Because there was a linear relationship between total VFA concentration treatment means and their SEM both in batch and continuous cultures (*p* < 0.001) that would have weighted down treatments with greater total VFA concentration, it was decided to use the reciprocal of the coefficient of variation (1/CV) of total VFA concentration as a weighting variable, instead of 1/SEM of total VFA concentration. The studies by Slyter and Wolin (1967) and Chalupa et al. (1980) did not report statistics of variation, and their treatment means were arbitrarily assigned a scaled weighting factor equal to one to maintain the original scale of the data.

Homoscedasticity was examined through the significance of the linear regression coefficient of residuals against predicted values (residual plots) and against the regressor *[2H]_CH4_*. Normality assumption was examined through residuals normality plots. Outliers and influential treatment means were identified by examining studentized residuals, leverage (hat) values, and Cook's distances. Outliers were identified as treatment means with a studentized residual > |*t_n − k, 0.95_*|, with *k* being the number of parameters and n the number of treatment means used to fit the regression. Influential treatment means were identified as those with a leverage value larger than 2*k/n* (Belsey et al., 1980) or a Cook's distance greater than the 50th percentile of an *F_k, n − k_* distribution.

Experiments containing treatment means so identified as outliers and/or influential observations were deleted one at a time, and regressions were fitted again in their absence. Treatment means were confirmed to be influential outliers if the conclusions of the analysis changed after the deletion of the experiment containing them, i.e., significant (*p* < 0.05) responses became non-significant (*p* > 0.15) or *vice versa*, or the direction of the response changed. When the presence of influential outliers was confirmed, results are presented both with and without the experiments containing the outliers.

### Factors affecting the responses to methanogenesis inhibition

When the interaction between *[2H]_CH4_* and the experiment effect was significant, the experiment effect was replaced by the following co-variables in order to understand possible causes of the variation among experiments in the responses to methanogenesis inhibition:

Batch cultures: ruminal fluid donor species (bovine or ovine), inoculum (ml), substrate (mg), percentage of concentrate in substrate, duration of incubation (h), and type of methanogenesis inhibitor;
Continuous cultures: substrate (g), percentage of concentrate in the substrate, liquid volume, fractional turnover rate of the liquid phase (h^−1^), and type of methanogenesis inhibitor.

Methanogenesis inhibitors were classified according to their known or presumed mode of action into: (i) Pure chemical compounds inhibiting or presumed to inhibit methanogens directly; (ii) nitrate and nitrocompounds, which apart from being toxic to methanogens can also decrease CH_4_ production by competing as electron acceptors for [H]; (iii) ionophores, which mainly inhibit organisms involved in producing H_2_; (iv) oils, which can inhibit methanogens directly, and protozoa that harbor them; (v) antiprotozoal agents; (vi) plant extracts. It is acknowledged that there is overlap between types of methanogenesis inhibitors as grouped herein, and more than one mechanism of action can apply to the same group of compounds, and that mechanisms of action of some inhibitors or additives are not understood. In order to allow analyzing experiments and treatments including more than one type of methanogenesis inhibitor, dummy variable columns were set in the batch and continuous culture databases, indicating, for each treatment mean, the presence or absence of each type of methanogenesis inhibitor.

The backward stepwise procedure was used to identify those co-variables whose interactions with *[2H]_CH4_* were potentially important to explain the responses:

(6) $$\begin{array}{l} response_{ij}=\text{intercept}+cov_{i}+{\text{B}}_{1}{\left[2H\right]}_{CH4j}+{\text{B}}_{2}{\left[2H\right]}_{CH4j}^{2}\\ \;+\;{\text{b}}_{i}{\left[2H\right]}_{CH4ij}+residual_{ij} \end{array}$$

Where *cov_i_* is the fixed effect of co-variable i, B_1_ and B_2_ are fixed linear and quadratic regression coefficients of *[2H]_CH4_*, respectively, and b*_i_* is the fixed effect of co-variable i on the linear *[2H]_CH4_* coefficient (i.e., interaction between co-variable *i* and methanogenesis inhibition). The quadratic effect of *[2H]_CH4_* was included only if it had not been removed (*p* < 0.15) from the original model including the experiment effect. Final models with the retained co-variables were selected by minimizing the corrected Akaike Information Criterion.

Substrates were classified into roughages (less than 33% concentrate in the dry matter), mixed (between 33 and 66% concentrate in the dry matter), and high concentrate (more than 66% concentrate in the dry matter). Regressions of *[2H]_Pr_, [2H]_But_* and *[2H]_H2_* against *[2H]_CH4_* including the interaction between *[2H]_CH4_* by type of substrate and the effect of the experiment were conducted jointly for five batch culture experiments that compared responses between a roughage and a mixed substrate (O'Brien et al., 2013). Experiments in continuous culture conducted with more than one type of substrate (Dong et al., 1997; Machmüller et al., 2001; Klevenhusen et al., 2009) could not be grouped for joint regressions because they were not orthogonal with regard to the type of substrate incubated. Results of regressions per type of substrate in continuous culture are presented for numerical comparison.

Also, the interaction of *[2H]_CH4_* with pH and dry matter apparent digestibility (DMD) on the response variables in batch cultures, and the interaction of *[2H]_CH4_* with pH, apparent digestibility of organic matter and neutral detergent fiber, and total bacterial and protozoal numbers in continuous culture, were evaluated. These regressions included the experiment effect and its interaction with *[2H]_CH4_*. In order to provide further insights on the effects of different inhibitors, inhibitors redirection of [H] toward propionate, butyrate and H2 was also numerically compared without adjusting for other co-variables.

In addition, responses to *[2H]_CH4_* in individual experiments were visually assessed, and experiments with a response different to the overall response were identified through the *p* value of the interaction between *[2H]_CH4_* and the particular experiment.

JMP® 11.0.0 (SAS Institute, Cary, NC, USA) was used for all statistical analyses.

## Results

Summaries of descriptive statistics for the batch and continuous culture databases are shown in Table 1. Descriptive statistics for main [H] sinks in control treatments, expressed as a percentage of *[2H]_produced_*, are shown in Table 2. There were no issues of heteroscedasticity in any regression. Most residuals normality plots were slightly, but not severely, S-shaped (not shown).

**Table 1:** **Summary of descriptive statistics^a^**.

**Variable**	**Batch culture**					**Continuous culture**				
	***N***	**number of experiments**	**mean**	***SD***	**min – max**	***N***	**number of experiments**	**mean**	***SD***	**min – max**
pH	88	10	6.39	0.29	5.70 – 6.70	66	12	6.69	0.38	5.13 – 7.20
Total gas production (ml or ml/d)	138	20	64.7	27.1	2.7 – 105	43	8	1028	606	76.7 – 2251
CO_2_ (ml/100 ml total gas)	67	13	77.8	8.62	61.8 – 97.2	43	8	88.5	5.43	74.7 – 98.7
CH_4_ (ml/100 ml total gas)	138^b^	20	10.1	9.10	ND^c^ – 42.4	43	8	10.5	6.07	ND – 25.2
H_2_ (ml/100 ml total gas)	138^b^	20	6.70	10.2	ND – 38.3	43	8	0.98	1.19	ND – 5.11
Total VFA^c^ (mM)	193	28	65.5	26.7	17.6 – 155	79	16	92.5	28.3	30.1 – 130
Acetate (molar%)	193	28	55.3	10.7	33.2 – 88.1	79	16	53.0	7.75	34.4 – 77.4
Propionate (molar%)	193	28	28.9	7.66	8.44 – 51.0	79	16	23.1	7.39	10.4 – 45.5
Butyrate (molar%)	193	28	12.0	5.97	1.47 – 32.4	79	16	15.7	4.91	5.70 – 27.1
Valerate (molar%)	103	16	2.59	1.24	0.29 – 6.59	61	13	5.42	2.62	0 – 10.0
Ac:Pr^c^ ratio (mol/mol)	193	28	2.16	1.37	0.84 – 10.4	79	16	2.61	1.15	0.95 – 6.41

a *Non-weighted treatment means*.

b *There are a total of 193 treatment means for CH_4_ and H_2_ production, however, the percentage of CH_4_ and H_2_ in total gas can only be calculated from their production for those experiments that reported CO_2_ or total gas production*.

c *Abbreviations: ND, not detected; Total VFA, total volatile fatty acids; Ac, acetate; Pr, propionate*.

**Table 2:** **Metabolic hydrogen sinks in control treatments^a^**.

	**Batch cultures**			**Continuous cultures**		
	**Mean**	***SD***	**Range**	**Mean**	***SD***	**Range**
*[2H]_CH4_* (%*[2H]_produced_*)^b^	46.6	12.8	12.6 – 86.1	29.1	11.6	10.0 – 64.6
*[2H]_Pr_* (%*[2H]_produced_*)	22.6	4.92	8.52 – 29.5	23.6	10.3	11.8 – 51.3
*[2H]_But_* (%*[2H]_produced_*)	11.3	4.45	2.76 – 21.3	15.5	4.07	7.5 – 22.9
*[2H]_H2_* (%*[2H]_produced_*)	0.043	0.086	0^c^ – 0.38	0.26	0.38	0^c^ – 1.65
*[H]_recovery_* (%)	80.5	17.0	41.6 – 121	68.5	13.3	52.9 – 105

a *Non-weighted treatment means*.

b *Abbreviations: [2H]_produced_, total amount of reducing equivalents pairs produced; [2H]_CH4_, [2H]_Pr_, [2H]_But_ and [2H]_H2_, percentage of metabolic hydrogen produced incorporated into CH_4_, propionate, butyrate and H_2_, respectively; [2H]_recovery_, percentage of hydrogen produced recovered in propionate, butyrate, H_2_ and CH_4_*.

c *^c^H_2_ reported as non-detected*.

**Figure 1:**
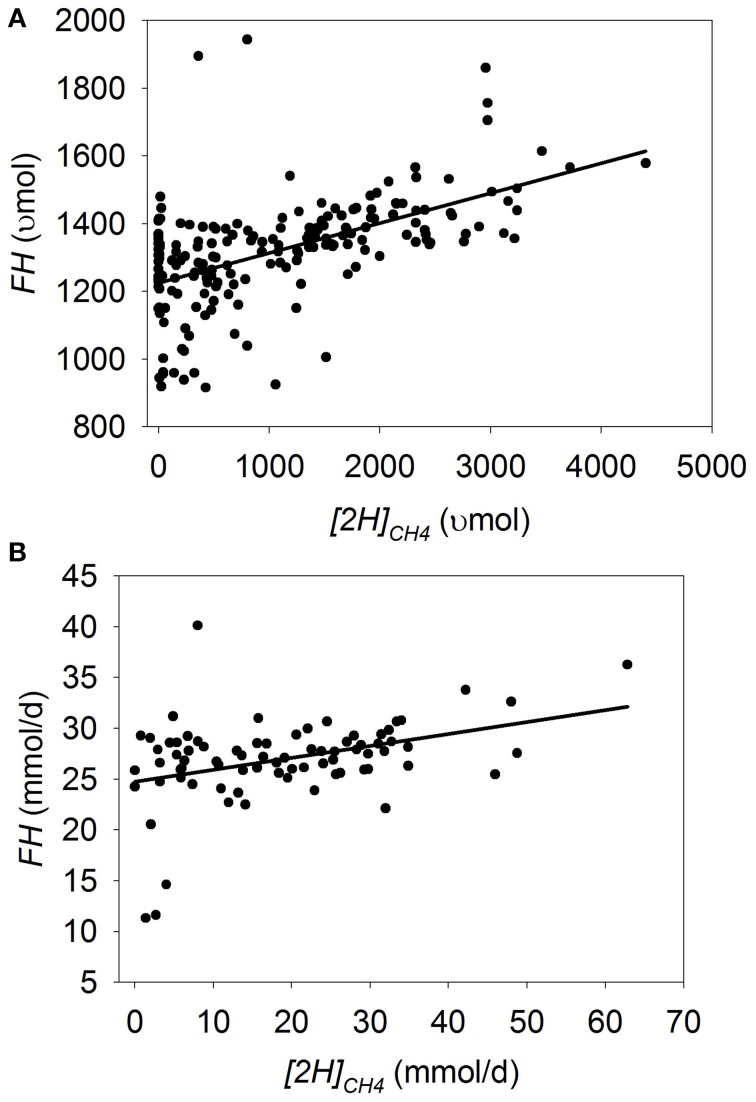
**Response of estimated fermented hexoses (*FH*) to metabolic hydrogen incorporated into CH_4_ (*[2H]_CH4_*)**. Individual responses are adjusted by their experiment effect (*exp*): **(A)** Batch cultures: *y* = 1186 (±44.6; *p* < 0.001) + *exp* (*p* < 0.001) + 0.11 (±0.068; *p* = 0.12) *x* + *exp* × *x* (*p* = 0.041); *R*^2^ = 0.94 (*p* < 0.001); **(B)** Continuous cultures: y = 24.4 (±0.82; *p* < 0.001) + *exp* (*p* < 0.001) + 0.16 (±0.033; *p* < 0.001) *x*; (*R*^2^ = 0.74; *p* < 0.001).

**Figure 2:**
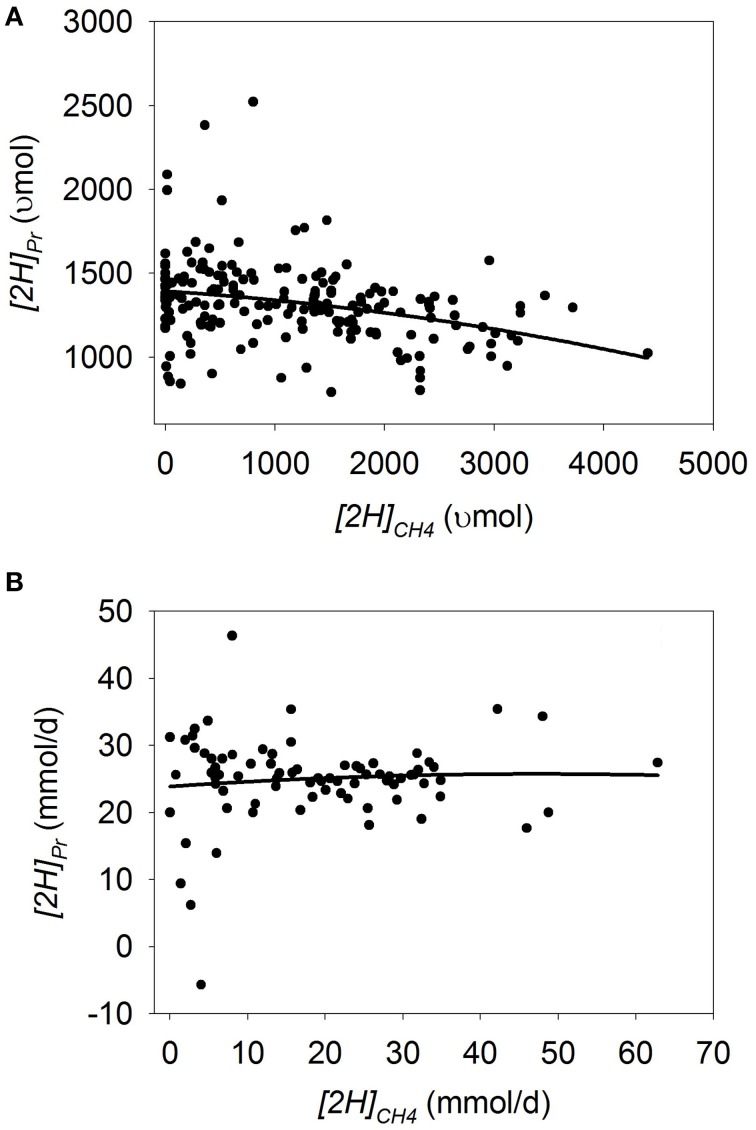
**Response of metabolic hydrogen incorporated into propionate (*[2H]_Pr_*) to metabolic hydrogen incorporated into CH_4_ (*[2H]_CH4_*)**. Individual responses are adjusted by their experiment effect (*exp*): **(A)** Batch cultures: *y* = 1443 (±42.0; *p* < 0.001) + *exp* (*p* < 0.001) – 0.079 (±0.018; *p* < 0.001) *x* – 2.63 × 10^−5^ (±1.74 × 10^−5^; *p* = 0.13) (*x* – 1366)^2^; *R*^2^ = 0.82 (*p* < 0.001); **(B)** Continuous cultures: *y* = 26.7 (±2.58; *p* < 0.001) + *exp* (*p* < 0.001) -0.040 (±0.14; *p* = 0.77) *x* – 0.0075 (±0.0050; *p* = 0.14) (*x* – 18.4)^2^ + *exp* × *x* (*p* = 0.08); R^2^ = 0.79 (*p* < 0.001).

**Figure 3:**
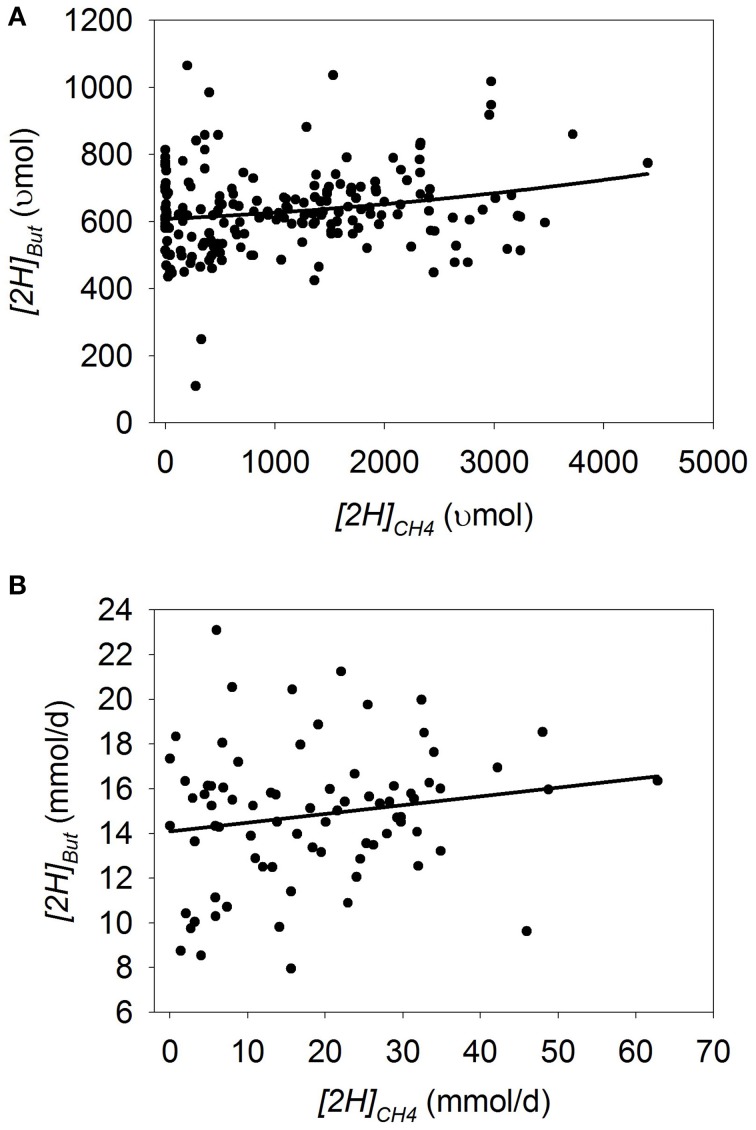
**Response of metabolic hydrogen incorporated into butyrate (*[2H]_But_*) to metabolic hydrogen incorporated into CH_4_ (*[2H]_CH4_*)**. Individual responses are adjusted by their experiment effect (*exp*): **(A)** Batch cultures: *y* = 556 (±42.4; *p* < 0.001) + *exp* (*p* < 0.001) + 0.0508 (± 0.061; *p* = 0.41) *x* + 1.94 × 10^−5^ (±1.29 × 10^−5^; *p* = 0.13) (*x* − 1366)^2^ + *exp* × *x* (*p* < 0.001); *R*^2^ = 0.93 (*p* < 0.001); **(B)** Continuous cultures: *y* = 14.4 (±1.05; *p* < 0.001) + *exp* (*p* < 0.001) + 0.012 (±0.056; *p* = 0.84) *x* + *exp* × *x* (*p* = 0.006); *R*^2^ = 0.87 (*p* < 0.001).

**Figure 4:**
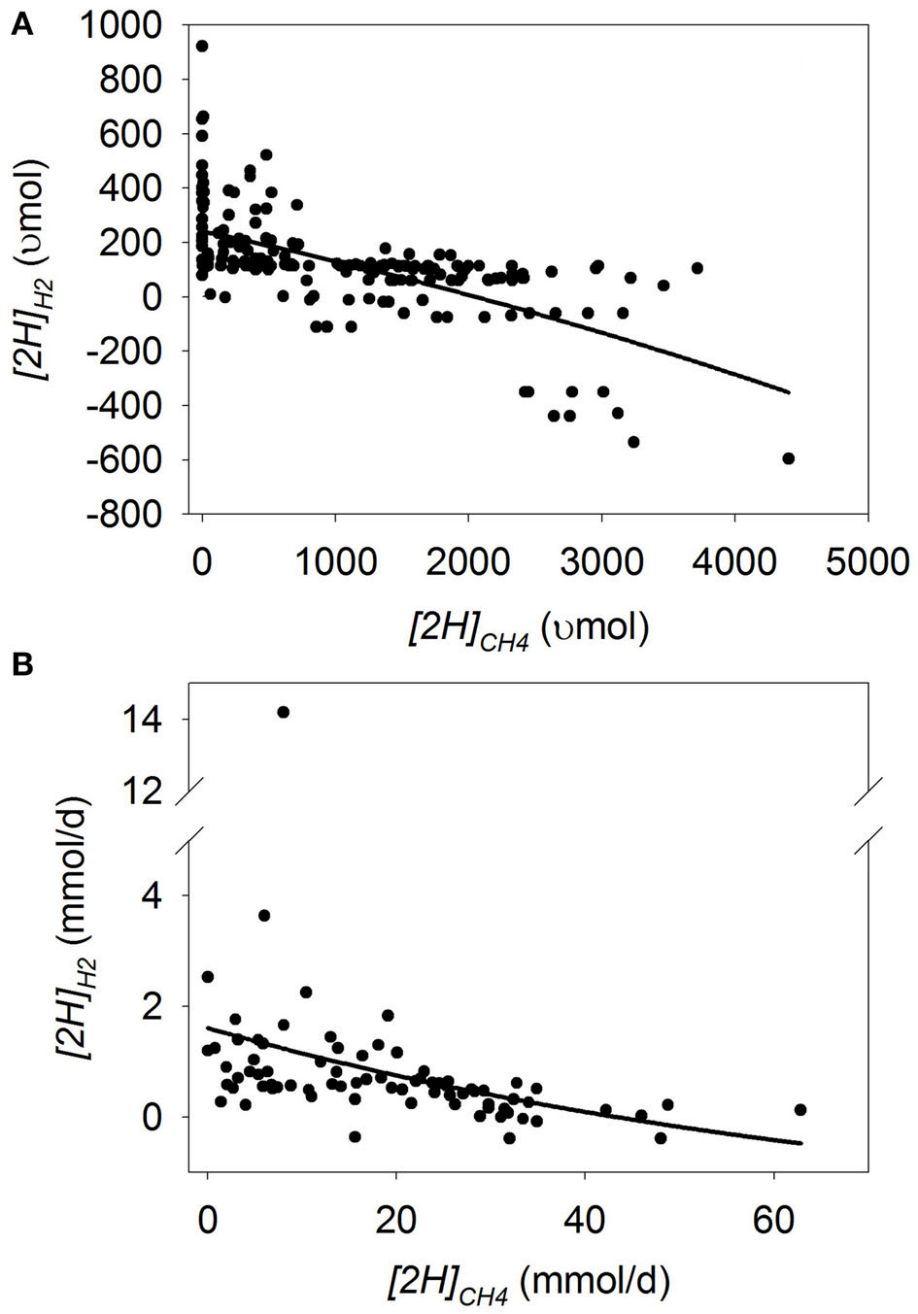
**Response of metabolic hydrogen incorporated into dihydrogen (*[2H]_H2_*) to metabolic hydrogen incorporated into CH_4_ (*[2H]_CH4_*)**. Individual responses are adjusted by their experiment effect (*exp*): **(A)** Batch cultures: *y* = 164 (±41.8; *p* < 0.001) + *exp* (*p* < 0.001)–0.0907 (±0.0607; *p* = 0.14) *x* + 5.77 × 10^−5^ (±1.27 × 10^−5^; *p* < 0.001) (*x* − 1366)^2^ + *exp* × *x* (*p* < 0.001); *R*^2^ = 0.91; *p* < 0.001; **(B)** Continuous cultures: L_N_ (*y* + 1) = 1.041 (±0.13; *p* < 0.001) + *exp* (*p* < 0.001) − 0.033 (±0.0070; *p* < 0.001) *x* + *exp* × *x* (*p* = 0.042); R^2^ = 0.76 (*p* < 0.001).

**Figure 5:**
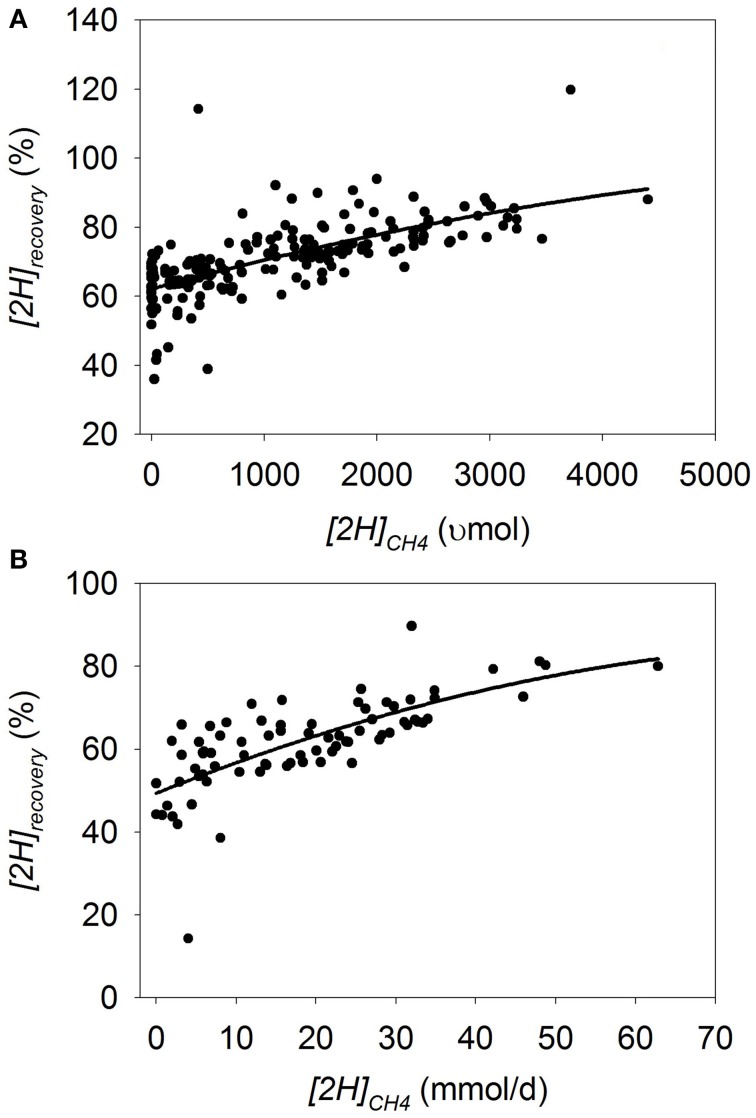
**Response of the percentage of metabolic hydrogen produced recovered in CH_4_, propionate, butyrate and H_2_ (*[2H]_recovery_*) to metabolic hydrogen incorporated into CH_4_ (*[2H]_CH4_*)**. Individual responses are adjusted by their experiment effect (*exp*): **(A)** Batch cultures: *y* = 59.7 (±1.76; *p* < 0.001) + *exp* (*p* < 0.001) + 0.0144 (±0.00255; *p* < 0.001) *x* − 1.12 × 10^−6^ (±5.32 × 10^−7^; *p* = 0.037) (*x* − 1366)^2^ + *exp* × *x* (*p* < 0.001); *R*^2^ = 0.91 (*p* < 0.001); **(B)** Continuous cultures: *y* = 51.0 (±2.30; *p* < 0.001) + *exp* (*p* < 0.001) + 0.64 (±0.12; *p* < 0.001) *x* − 0.015 (±0.0045; *p* = 0.002) (*x* − 18.4)^2^ + *exp* × *x* (*p* = 0.017); *R*^2^ = 0.87 (*p* < 0.001).

**Figure 6:**
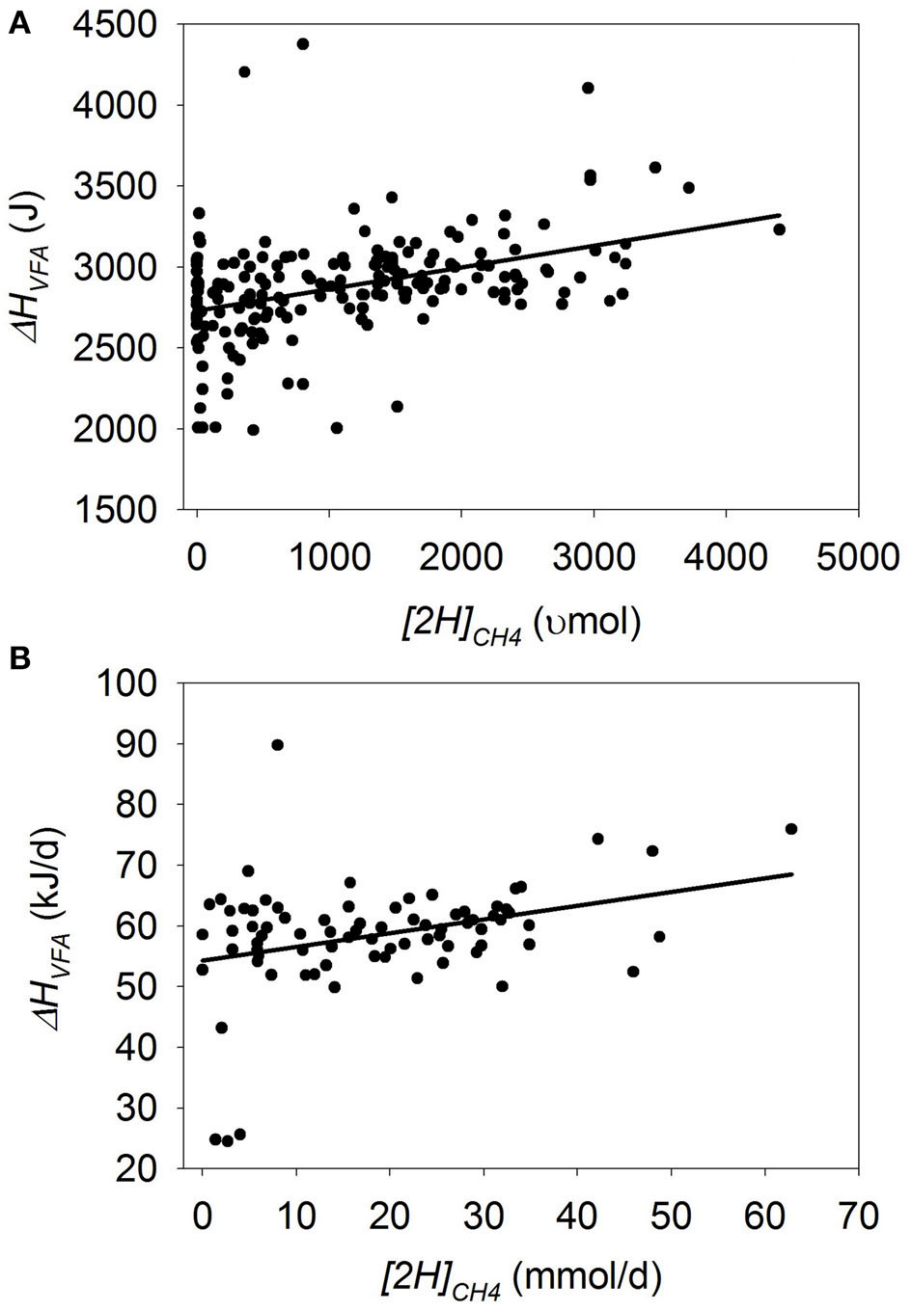
**Response of the heat of combustion in volatile fatty acids (Δ*H_VFA_*) to metabolic hydrogen incorporated into CH_4_ (*[2H]_CH4_*)**. Individual responses are adjusted by their experiment effect (*exp*): **(A)** Batch cultures: *y* = 2664 (±103; *p* < 0.001) + *exp* (*p* < 0.001) + 0.162 (±0.156; *p* = 0.30) *x* + *exp* × *x* (*p* = 0.06); R^2^ = 0.93 (*p* < 0.001); **(B)** Continuous cultures: *y* = 53.9 (±1.91; *p* < 0.001) + *exp* (*p* < 0.001) + 0.29 (±0.077; *p* < 0.001) *x*; R^2^ = 0.72 (*p* < 0.001).

**Figure 7:**
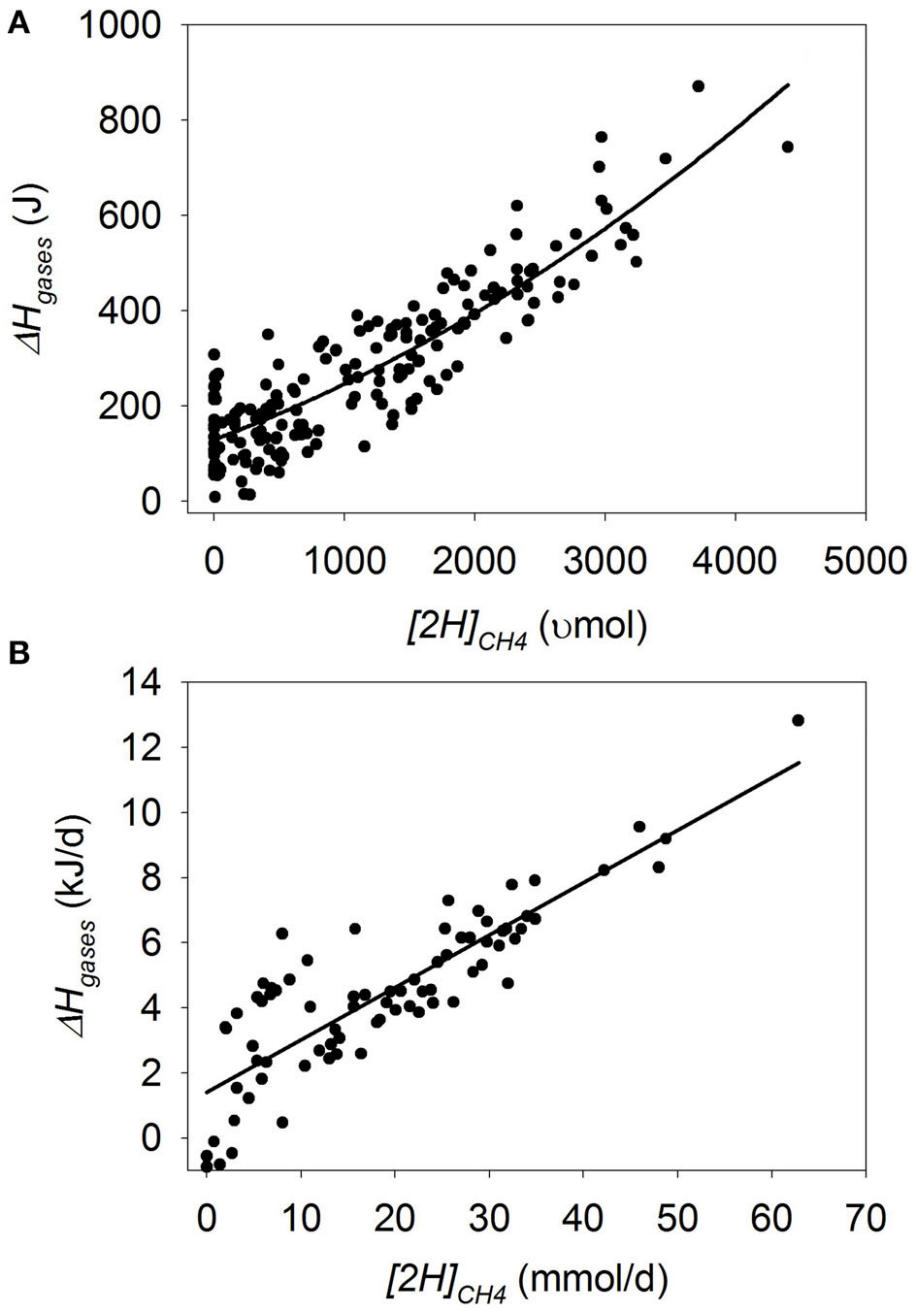
**Response of the heat of combustion in gases (Δ*H_gases_*) to metabolic hydrogen incorporated into CH_4_ (*[2H]_CH4_*)**. Individual responses are adjusted by their experiment effect (*exp*): **(A)** Batch cultures: *y* = 46.9 (±12.0; *p* < 0.001) + *exp* (*p* < 0.001) + 0.20 (±0.017; *p* < 0.001) *x* + 1.65 × 10^−5^ (±3.63 × 10^−6^; *p* < 0.001) (*x* − 1366)^2^ + *exp* × *x* (*p* < 0.001); *R*^2^ = 0.98 (*p* < 0.001); **(B)** Continuous cultures: *y* = 0.66 (±0.15; *p* < 0.001) + *exp* (*p* = 0.057) + 0.21 (±0.0062; *p* < 0.001) *x*; *R*^2^ = 0.96 (*p* < 0.001).

Plots of [H] sinks as a function of *[2H]_CH4_* are adjusted by the experiment effect (Figures 1–**7**). In some plots, adjusting by the experiment effect caused some treatment means with low values for the response variable belonging to an experiment with a high positive experiment effect to have negative experiment-adjusted responses.

### Fermentation and digestion

In batch cultures, there was an interaction between *[2H]_CH4_* and the experiment effect on *FH* (*p* = 0.041; Figure 1A), with a tendency to a decrease in *FH* with the decrease in *[2H]_CH4_* (i.e., methanogenesis inhibition; *p* = 0.12). Excluding the experiment by Anderson et al. (2010), in which inhibiting methanogenesis was associated with greater *FH*, resulted in a linear decrease in *FH* with the decrease in *[2H]_CH4_* (*p* < 0.001; not shown) with an interaction with the experiment effect (*p* = 0.044). Greater inoculum volume (*p* = 0.001) and chemical inhibitors of methanogenesis (*p* = 0.001) were associated with greater inhibition in *FH* as *[2H]_CH4_* decreased, in contrast to greater concentrate percentage in the substrate (*p* = 0.003; not shown). Use of chemical inhibitors was in turn positively associated with inoculum volume (*p* < 0.001), which may have caused the unexpected negative association between inoculum volume and *FH*. There was an interaction between *[2H]_CH4_* and the experiment effect on DMD (*p* < 0.001), with decreases (*p* ≤ 0.03) or tendencies (*p* < 0.14) to decrease DMD with methanogenesis inhibition in three experiments, increases (*p* < 0.003) or tendencies (*p* < 0.09) to increase in two, and lack of effects in one (*p* = 0.87; not shown).

In continuous cultures, there was a linear decrease in *FH* as *[2H]_CH4_* decreased (*p* < 0.001), with no interaction with the experiment effect (*p* = 0.40; Figure 1B). There was a tendency to decrease apparent digestibility of organic matter (*p* < 0.07), and a decrease in neutral detergent fiber digestibility (*p* < 0.001) with the decrease in *[2H]_CH4_*, with an interaction with the experiment effect (*p* = 0.009; not shown).

### Propionate

In batch cultures, there was a an increase in *[2H]_Pr_* as methanogenesis was inhibited (*p* < 0.001) with a tendency to be quadratic (*p* = 0.13; Figure 2A; Table 3) and no interaction with the experiment effect (*p* = 0.26). In the five experiments in the study by O'Brien et al. (2013), inhibiting methanogenesis resulted in greater increase in *[2H]_Pr_* with a mixed than with a roughage substrate (*p* < 0.001; Table S3).

**Table 3:** **Metabolic hydrogen balance predicted^a^ for 0 (control treatments)^b^ and 100% methanogenesis inhibition**.

	***[2H]_produced_*^c^ (μmol or mmol/d)**	**Metabolic hydrogen in:**				***[2H]_recovery_* (%)^c^^,^^e^**
		**CH_4_ (μmol or mmol/d)**	**Propionate (μmol or mmol/d)**	**Butyrate (μmol or mmol/d)**	**H_2_^d^ (μmol or mmol/d)**	
**BATCH CULTURES**						
Control treatments	5248	2570	1201	715	14.4	95.2
100% methanogenesis inhibition	4033	0	1394	592	272	57.6
**CONTINUOUS CULTURES**						
Control treatments	103	29.0	24.7	14.6	0.087	67.9
100% methanogenesis inhibition	85.5	0	24.2	14.3	1.83	46.1

a *Regression equations for metabolic hydrogen incorporated in propionate, butyrate, H_2_ and metabolic hydrogen recovery are provided in Figures 2, 3, 4, 5, respectively*.

b *Weighted percentage of [2H]_produced_ incorporated into CH_4_ in non-inhibited control treatments was 45.5 and 28.4% for batch and continuous cultures, respectively*.

c *Abbreviations: [2H]_produced_, total production of reducing equivalents pairs; [2H]_recovery_, sum of reducing equivalents pairs incorporated into CH_4_, propionate, butyrate and H_2_ divided by [2H]_produced_, expressed as percentage*.

d *For continuous cultures, metabolic hydrogen incorporated into H_2_ is presented backtransformed*.

e *Because [2H]_recovery_ is the quotient between treatment means for [2H]_incorporated_ and [2H]_produced_ expressed as a percentage, predicted values for [2H]_recovery_ are not equal to the quotient of predicted value of [2H]_incorporated_ divided by predicted values of [2H]_produced_ expressed as a percentage*.

In continuous cultures, there was a quadratic tendency to a decrease in *[2H]_Pr_* with methanogenesis inhibition (*p* = 0.14), with a tendency to an interaction with the experiment effect (*p* = 0.08; Figure 2B; Table 3). Chemical inhibitors of methanogenesis (*p* = 0.02), oils (*p* = 0.09) and greater liquid volume (*p* = 0.10) associated or tended to associate with greater [H] redirection toward propionate when methanogenesis was inhibited.

Both in batch culture and continuous culture, ionophores and linoleic and linolenic acids were numerically associated with a greater response in *[2H]_Pr_* than other types of inhibitors. In continuous culture, cashew nut shell liquid resulted in the numerically greater response in *[2H]_Pr_* when inhibiting methanogenesis, although these results were provided by one single experiment (Table S4).

### Butyrate

In batch cultures, there was an interaction between *[2H]_CH4_* and the experiment effect on *[2H]_But_* (*p* < 0.001), with an overall quadratic tendency to decrease *[2H]_But_* with methanogenesis inhibition (*p* = 0.13; Figure 3A; Table 3). Ionophores (*p* = 0.007) associated with greater decrease in *[2H]_But_* with methanogenesis inhibition, whereas greater concentrate percentage (*p* = 0.016) and chemical inhibitors (*p* < 0.001) associated with lesser decrease in *[2H]_But_* (not shown). There was a tendency (*p* = 0.13) toward less decrease in *[2H]_But_* with methanogenesis inhibition with a mixed than with a roughage substrate in the five experiments of the study by O'Brien et al. (2013) (Table S3).

In continuous cultures, there was an interaction between *[2H]_CH4_* and the experiment effect on *[2H]_But_*(*p* = 0.006), with no overall relationship between *[2H]_But_* and *[2H]_CH4_* (*p* = 0.84; Figure 3B; Table 3). Removing the experiment by García-López et al. (1996) from the analysis resulted in a decrease in *[2H]_But_* with the decrease in *[2H]_CH4_* (*p* = 0.032; not shown). Greater turnover time (*p* = 0.015) and concentrate percentage (*p* = 0.024), and use of chemical inhibitors of methanogenesis (*p* < 0.001), positively associated with *[2H]_But_* as methanogenesis was inhibited (not shown).

### Dihydrogen

In batch cultures, there was a quadratic increase in *[2H]_H2_* with *[2H]_CH4_* decrease (*p* < 0.001), with an interaction with the experiment effect (*p* < 0.001; Figure 4A; Table 3). Greater amount of substrate (*p* = 0.034), concentrate percentage (*p* < 0.001), and duration of incubation (*p* < 0.08) associated or tended to associate with increased response in *[2H]_H2_* to *[2H]_CH4_* decrease. Ionophores (*p* = 0.038) and oils (*p* < 0.001) associated with lesser response in *[2H]_H2_* to *[2H]_CH4_* decrease (not shown). There was a tendency (*p* = 0.06) toward greater increase in *[2H]_H2_* with methanogenesis inhibition with a mixed than with a roughage substrate in the study by O'Brien et al. (2013) (Table S3).

In continuous culture, natural log transformation of the response variable *[2H]_H2_* was necessary to partially correct for a funnel-shaped residual plot. There was a linear increase in natural log *[2H]_H2_* with the decrease in *[2H]_CH4_* (*p* < 0.001) with an interaction with the experiment effect (*p* = 0.042; Figure 4B; Table 3). There was no interaction if the experiment by García-López et al. (1996), with the greatest H_2_ accumulation, was eliminated from the analysis (*p* = 0.22; not shown). Greater concentrate percentage in the substrate was associated to greater *[2H]_H2_* when methanogenesis was inhibited (*p* = 0.031; not shown).

Linoleic and linolenic acid, and nitrate and nitrocompounds in batch culture, and ionophores in continuous culture, were associated with the numerically smallest increase in *[2H]_H2_* with methanogenesis inhibition (Table S4).

### *[2H]*_recovery_

In batch cultures, there was a quadratic (*p* = 0.037) decrease in *[2H]_recovery_* as *[2H]_CH4_* decreased (*p* < 0.001) with an interaction *[2H]_CH4_* by experiment (*p* < 0.001; Figure 5A; Table 3). Bovine inoculum (*p* < 0.001) and greater inoculum volume (*p* < 0.001) were associated with greater decrease in *[2H]_recovery_* with methanogenesis inhibition. On the contrary, the decrease in *[2H]_recovery_* with methanogenesis inhibition was smaller with greater amount of substrate (*p* = 0.001), greater concentrate percentage (*p* = 0.006), and if CH_4_ production was inhibited with oils (*p* < 0.001; not shown).

In continuous cultures, there was also a quadratic (*p* = 0.002) decrease in *[2H]_recovery_* with methanogenesis inhibition (*p* < 0.001) with an interaction with the experiment effect (*p* = 0.017; Figure 5B; Table 3). Greater concentrate percentage (*p* = 0.04) resulted in less *[2H]_recovery_* when methanogenesis was inhibited, whereas the use of oils to inhibit methanogenesis resulted in less decrease in *[2H]_recovery_* (*p* = 0.002; not shown).

### Heat of combustion in VFA

In batch cultures, there was no overall response in Δ*H_VFA_* to methanogenesis inhibition (*p* = 0.30), although there was a tendency for an interaction between *[2H]_CH4_* and the experiment effect (*p* = 0.06; Figure 6A). If the experiment by Anderson et al. (2010) was removed, in which *FH* increased with methanogenesis inhibition, there was a negative association between Δ*_HVFA_* and methanogenesis inhibition (*p* = 0.016; not shown). Greater concentrate percentage (*p* < 0.001), duration of incubation (*p* < 0.001) and utilization of chemical inhibitors of methanogenesis (*p* < 0.001) all interacted positively with Δ*H_VFA_* when methanogenesis was inhibited (not shown).

In continuous cultures, inhibiting methanogenesis associated with a decrease in Δ*H_VFA_* (*p* < 0.001), with no interaction with the experiment effect (*p* = 0.52; Figure 6B). However, there was no association of methanogenesis inhibition with Δ*H_VFA_* after eliminating from the analysis the experiment by Slyter (1979), which contained two outliers in which methanogenesis inhibition was caused by lowering *pH* (*p* = 0.38; not shown), or the experiment by Slyter and Wolin (1967) (*p* = 0.27; not shown).

### Heat of combustion in gases

In batch cultures, there was a quadratic decrease in Δ*H_gases_* when methanogenesis was inhibited (*p* < 0.001), with an interaction with the experiment effect (*p* < 0.001; Figure 7A). Greater amount of substrate (*p* = 0.034), concentrate percentage (*p* < 0.001) and duration of the incubation (*p* = 0.08), associated or tended to associate with smaller decrease in Δ*H_gases_* as methanogenesis was inhibited. On the contrary, utilization of ionophores (*p* = 0.038) and oils (*p* < 0.001) associated with greater decrease in Δ*H_gases_* with methanogenesis inhibition.

In continuous cultures, there was a linear decrease in Δ*H_gases_* with methanogenesis inhibition (*p* < 0.001), with no interaction with the experiment effect (*p* = 0.46; Figure 7B).

## Discussion

### Metabolic hydrogen recovery and missing sinks

A complete [H] balance for predicted 0 and 100% methanogenesis inhibition in batch and continuous cultures is shown in Table 3. In batch cultures, at predicted 100% methanogenesis inhibition, an average of only 0.075 mol [H] per mol of [H] drawn away from CH_4_ formation were redirected to propionate production (calculations from Table 3), and there was a tendency to less [H] into butyrate. There was no overall re-direction of [H] toward propionate and butyrate in continuous culture when methanogenesis was inhibited (Table 3). Inhibiting methanogenesis caused accumulation of H_2_ (Figures 4A,B), but H_2_ accumulation at predicted 100% methanogenesis inhibition accounted for only 10 and 6.0% of [H] in CH_4_ in control treatments in batch and continuous cultures, respectively. There was less production of [H] when methanogenesis was inhibited both in batch and continuous cultures (Table 3); however, even when accounting for less *[H]_produced_, [2H]_recovery_* in the main fermentation products decreased severely both in batch and continuous cultures when methanogenesis was inhibited (Figures 5A,B; Table 3). Therefore, a major [H] sink or sinks in the methanogenesis-inhibited ruminal fermentation was unaccounted for. Three potential unaccounted [H] sinks will be discussed: (i) fermentation products other than propionate, butyrate and H_2_, (ii) microbial biomass, and (iii) reductive acetogenesis.

#### Other products of fermentation

Most experiments used in this meta-analysis reported valerate production. In batch cultures, the participation of valerate as a [H] sink slightly decreased from 4.8 to 4.5% between predicted 0 and 100% methanogenesis inhibition (not shown). In continuous cultures, valerate increased its participation as a [H] sink from 7.9 to 10.7% of total *[2H]_produced_* between predicted 0 and 100% methanogenesis inhibition (not shown). Caproate is an end product of fermentation even more reduced than valerate. Caproate was reported only in the study by Anderson et al. (2010) in batch culture, and tended to increased its participation from 2.2 to 4.8% of total *[2H]_produced_* between predicted 0 and 100% methanogenesis inhibition (not shown). Formate was reported in only 6 batch culture experiments, and at predicted 100% methanogenesis inhibition it accounted for an average of 16% of [H] in CH_4_ in the corresponding control treatments (not shown). This figure is close to the missing [H] in CH_4_ found in formate in recent experiments, which agree with the estimation obtained by Hungate et al. (1970) of 18% ruminal CH_4_ formed from formate (S. Muetzel, pers.com). Immig (1996) reported no alterations in major fermentation products when formate was added to methanogenesis-inhibited incubations, suggesting that formate accumulated and was not further metabolized. In the continuous culture experiment by Slyter (1979), there was a slight formate accumulation when methanogenesis was inhibited using dichloroacetamide which accounted for little *[2H]_incorporated_*. Metabolic hydrogen incorporation into succinate, ethanol and lactate accounted for little *[2H]_incorporated_* both in batch and continuous cultures (not shown). It is important that formate and other atypical reduced end products of fermentation, apart from H_2_, as well as the more reduced VFA valerate and caproate, are reported in experiments in which methanogenesis is inhibited.

#### Microbial biomass

Czerkawski (1986) proposed that inhibiting methanogenesis could favor microbial biomass production as an alternative [H] sink. Chalupa (1977) suggested that [H] incorporated into excess NADH was redirected to fatty acids synthesis and fermentation end products such as lactate and ethanol, although the latter sinks were not quantitatively important in the experiments that reported them in the present analysis (not shown).

Microbial biomass production depends on the rates and efficiencies of ATP generation (catabolism) and utilization for growth (anabolism). Inhibiting methanogenesis decreases the system's reducing potential through greater availability of reducing equivalents in electron donors such as H_2_ (Sauer and Teather, 1987) and NADH (Hino and Russell, 1985). If anabolic processes that incorporate reducing equivalents, such as fixation of NH^+^_4_ into carbon chains for amino acids synthesis, and synthesis of fatty acids, were kinetically limited by the availability of [H] (or were close to thermodynamic equilibrium), increased availability of [H] consequence of inhibiting methanogenesis could stimulate amino acids and fatty acids synthesis and therefore microbial growth. Stimulation of microbial anabolism could then increase the importance of microbial biomass as a [H] sink through two mechanisms: (i) Greater microbial biomass production; (ii) A more reduced microbial biomass composition: if microbial content of lipids increased, that would mean that more [H] was incorporated per gram of microbial organic matter produced.

Inhibiting methanogenesis could also have consequences on microbial ATP generation. In anaerobic systems like the rumen, part of the negative Gibbs energy change associated to fermentation is used to generate ATP through substrate level and electron transport-linked phosphorylation. An inhibition of *FH*, as in most batch culture experiments in the present meta-analysis, should decrease ATP generation. Apart from being affected by *FH*, ATP generation can be affected by the fermentation profile. It has been shown in experiments with defined cultures that the presence of methanogens alters the pattern of fermentation of bacteria and fungi. A shift from acetate, ethanol, H_2_ and CO_2_ produced by *Ruminococcus albus* in pure culture, to acetate and CH_4_ in co-culture with a methanogen, increased ATP generation by *R. albus* and added the ATP generated by the methanogen (Wolin et al., 1997). Similarly, ruminal anaerobic fungi decreased the production of ethanol and lactate, and increased the production of acetate, when grown with methanogens, increasing ATP generation (Bauchop and Mountfort, 1981; Marvin-Sikkema et al., 1990).

However, when inhibiting methanogenesis in mixed cultures, accumulation of ethanol seems of small importance compared to pure cultures of ethanol producers. In the present meta-analysis, there was an increase in [H] incorporated into propionate production in batch cultures when methanogenesis was inhibited. This agrees with greater production of succinate and propionate by *R. flavefaciens* or *Selenomonas ruminantium*, respectively, when grown in pure culture than when co-cultured with methanogens (Wolin et al., 1997). It is important to understand what could be the consequences of an acetate to propionate shift on microbial ATP generation.

Propionate is produced *via* succinate (randomizing pathway) or acrylate (non-randomizing pathway) in the rumen. Reduction of fumarate to succinate is associated with ATP generation through a transmembrane electrochemical gradient (Russell and Wallace, 1997), but the acrylate pathway does not seem to be associated with ATP generation (Thauer et al., 1977). The proportions of extra propionate produced through the succinate and acrylate pathways when inhibiting methanogenesis are unknown, and therefore constitute an uncertainty of the consequences of an acetate to propionate shift upon ATP generation. Furthermore, the stoichiometry of ATP generation per pair of reducing equivalents incorporated in the reduction of fumarate to succinate is not clearly established as measurements have disagreed (Reddy and Peck, 1978; Kröger and Winkler, 1981).

Generation of ATP in propionate's randomizing pathway is also affected by other metabolic steps. Oxaloacetate (or malate) formation from phosphoenolpyruvate (PEP) entails the utilization of PEP high-energy phosphate bond, which is then not used to generate ATP, but when catalyzed by PEP carboxykinase, PEP carboxylation can still generate ATP or GTP without additional energy input (Atwal and Sauer, 1974). Also, when the entire randomizing pathway takes place in a single cell (i.e., without interspecies transfer of succinate), carboxylation of pyruvate to form oxaloacetate can occur coupled to methylmalonyl-CoA decarboxylation to propionyl-CoA, making oxaloacetate formation from pyruvate energetically neutral (Deborde and Boyaval, 2000).

Therefore, our knowledge about ATP generation by the mixed ruminal microbiota is incomplete due to the difficulties to determine ATP generation associated to propionate production. For the moment, it seems that we will have to rely on the few existing empirical results on the response of microbial growth to methanogenesis inhibition rather than on a deeper mechanistic understanding on the consequences of methanogenesis inhibition on microbial ATP generation and utilization. Improved microbial growth efficiency allowed obtaining increased microbial biomass with methanogenesis inhibition even with decreased true organic matter digestibility (Ungerfeld et al., 2007). Other *in vitro* work also found stimulation of microbial growth by methanogenesis inhibition (van Nevel et al., 1969; Guo et al., 2009). Nolan et al. (2010) reported a numerical increase of 27% in microbial protein production in sheep whose CH_4_ production was decreased by 23%. However, other work has found no effect or even a decrease in microbial growth with methanogenesis inhibition (van Nevel et al., 1969, 1971; Russell and Martin, 1984; Lee et al., 2009). Apart from biomass accretion, a more reduced microbial biomass composition could account for part of the “missing [H].” Changes in microbial biomass amount and composition, shifts in anabolic pathways, and ultimately ATP generation and utilization, as a response to methanogenesis inhibition, need to be studied.

#### Reductive acetogenesis

If some acetate produced when methanogenesis is inhibited was originated from reductive acetogenesis, part of acetate production would be an unaccounted [H] sink. In the typical ruminal fermentation, methanogens drop H_2_ pressure to a level at which reductive acetogenesis has been estimated to be thermodynamically unfeasible (Kohn and Boston, 2000) and as a result the process does not occur (le Van et al., 1998). However, reductive acetogenesis has been shown to occur in batch cultures if methanogenesis is inhibited and reductive acetogens are added (Nollet et al., 1997; le Van et al., 1998). Native reductive acetogens do inhabit the rumen even when methanogenesis is not inhibited (Leedle and Greening, 1988; Henderson et al., 2010), but they seem to rely on substrates other than CO_2_ and H_2_ (Joblin, 1999). It is possible that reductive acetogenesis could over time become a [H] sink in continuous culture and *in vivo* when methanogenesis is inhibited. It is unknown whether or not some reductive acetogens can adapt to methanogenesis-inhibited continuous culture conditions to conduct reductive acetogenesis. In the experiments used for this meta-analysis, acetate produced through fermentation of carbohydrates would be indistinguishable from acetate produced from reductive acetogenesis, if the latter occurred. Therefore, decreases observed in acetate production with methanogenesis inhibition (data not shown) are not evidence against the possibility of reductive acetogenesis.

### Variation in responses of [H] sinks to methanogenesis inhibition

Shifts in [H] sinks when methanogenesis was inhibited varied between batch and continuous cultures, and among experiments within each kind of system. It is of much interest to understand the causes of this variation, especially what the experiments with a greater than average response in *[2H]_Pr_* or *[2H]_But_*, and lower than average response in *[2H]_H2_*, have in common. Some factors that may affect [H] shifts when methanogenesis is inhibited are discussed below.

#### Batch vs. continuous cultures

Redirection of [H] toward propionate production when methanogenesis was inhibited was more favored in batch than in continuous cultures. Perhaps, important propionate producers do not survive well, or at least are not stimulated as much by methanogenesis inhibition, in continuous compared to batch culture. Decline in microbial diversity over time in continuous cultures has been reported (Johnson et al., 2009). For example, Broudiscou et al. (2014) reported that the effect of H_2_ pressure on propionate production per mol of *FH* in batch cultures depended on the source of inoculum, which were continuous cultures incubated with different substrates. Because they studied the effect of added H_2_ on short-term, 6 h batch incubations, the presence of sufficient numbers of propionate producers in their continuous culture inocula may have determined the response of propionate to H_2_. In fact, the greatest response in propionate to headspace H_2_ in the study by Broudiscou et al. (2014) came from the inoculum obtained from a continuous culture fed a roughage substrate which produced more propionate per mole of *FH* than two other continuous cultures fed different high concentrate substrates.

It is possible that some propionate or succinate producers do not adapt well to continuous cultures to take advantage of the favorable thermodynamic conditions for propionate production occurring when methanogenesis is inhibited. Differences between batch and continuous cultures have been reported for some succinate-producing bacteria with regard to their response to methanogenesis inhibition. For example, inhibiting methanogenesis resulted in an increase in *Fibrobacter succinogenes* in ruminal batch cultures (Guo et al., 2007, 2008; Goel et al., 2009), but no changes in continuous cultures (Goel et al., 2009), although lack of effects in batch cultures has also been reported (González et al., 2006). *R. flavefaciens* was either decreased (Goel et al., 2009) or unaffected (González et al., 2006; Guo et al., 2007, 2008) by inhibiting methanogenesis in batch cultures, and decreased in continuous cultures (Goel et al., 2009).

An alternative explanation to greater response in propionate in batch than in continuous culture could be based on thermodynamics. Most batch culture systems operate with pressurized tubes or bottles, allowing greater H_2_ pressure at equal H_2_ molar percentage in comparison to continuous cultures, where gas is collected at nearly atmospheric pressure. Elevation of H_2_ pressure favors a shift from acetate to propionate production (Janssen, 2010). Based on this technical difference between most batch and continuous cultures, it could be expected that batch cultures favor more a shift to propionate compared to continuous cultures. Indeed, there was a negative quadratic relationship between the estimated final H_2_ pressure in batch cultures and the acetate to propionate molar ratio (*R*^2^ = 0.62; *P* < 0.001; not shown). Theoretically, batch incubation systems that allow the release of gas to the atmosphere (Wang et al., 2013) should better mimic the *in vivo* situation from a thermodynamics point of view.

Despite most batch culture systems resulting in supra-atmospheric total gas pressures, inhibition of methanogenesis resulted in greater H_2_ accumulation in batch than in continuous culture. This could suggest some adaptation over time of continuous cultures to incorporate part of the accumulated H_2_ into other pathways not modeled in the present analysis e.g., reductive acetogenesis. In agreement, a progressive decrease in H_2_ accumulation in batch cultures from 24 to 48 and 72 h of incubation was observed when methanogenesis was inhibited with 2-bromoethanesulphonic acid (Lee et al., 2009). Still, mid- and long-term H_2_ accumulation has been reported to occur in continuous culture with high-concentrate substrates even in the absence of methanogenesis inhibitors (Broudiscou et al., 2014), as well as in various methanogenesis inhibition *in vivo* experiments (Trei et al., 1972; Clapperton, 1974; Kung et al., 2003; van Zijderveld et al., 2011; Mitsumori et al., 2012), and incubations of rumen contents from animals administered methanogenesis inhibitors for long periods (Czerkawski and Breckenridge, 1975). Therefore, it seems that, although there can be some long term adaptation of microbiota to incorporate [H] into alternative pathways in the methanogenesis-inhibited ruminal fermentation, this is not complete and consistent, and H_2_ remains as an end product of fermentation (with the possible exception of the use of some lipids as methanogenesis inhibitors, as discussed below).

It is difficult to conclude on whether the batch or continuous culture system might better reflect changes in fermentation occurring *in vivo* when methanogenesis is inhibited. If there is some long-term adaptation of microbiota to incorporate H_2_ into other pathways, the continuous culture systems may better represent the *in vivo* situation in comparison to batch culture. On the other hand, the slight overall increase in propionate production observed in batch culture may better reflect the increase in propionate concentration or molar proportion of most experiments when methanogenesis is inhibited *in vivo* (Clapperton, 1974; Davies et al., 1982; McCrabb et al., 1997; Abecia et al., 2012, 2013; Mitsumori et al., 2012), although actual VFA production, which includes VFA removed by both absorption and passage from the rumen, as well as VFA incorporated into microbial fatty acids and amino acids (Kristensen, 2001), was not quantified in those *in vivo* studies. Simultaneous *in vivo* measurement of VFA and gases actual production when inhibiting methanogenesis would be necessary to conclude which type of *in vitro* system, if any, can better mimic the *in vivo* situation.

#### Type of substrate

Both in batch and continuous culture, redirection of [H] toward H_2_ was greater with more concentrate in the substrate, and in batch culture there was greater H_2_ accumulation with greater DMD (not shown). Bauchop (1967) found a 2-fold increase in H_2_ accumulation when inhibiting methanogenesis with chlorinated CH_4_ analogs in the absence of added substrate, whereas H_2_ accumulation was much greater if ruminal solids or formate were incubated. Czerkawski and Breckenridge (1975) reported that when CH_4_ production was inhibited, H_2_ accumulation was much greater 2 h after the morning feeding, in an actively fermenting rumen, compared to pre-feeding levels. Perhaps, that could be an indication that incorporation of [H] into sinks other than H_2_ when fermentation is very active might be limited by the activity of alternative [H]-incorporating pathways i.e., enzyme kinetics. In steers not subjected to methanogenesis-inhibition treatments, Rooke et al. (2014) determined that animals fed a high concentrate diet produced gas with a greater ratio of H_2_ to CH_4_ compared to those fed a mixed diet, and suggested that rapid H_2_ production immediately after feeding can result in methanogens being overloaded in their capacity to utilize H_2_. This effect would likely accentuate in the methanogenesis-inhibited fermentation. Slowly degradable diets and uniform feed intake might then be strategies to decrease H_2_ accumulation when methanogenesis is inhibited. In that regard, Swainson et al. (2011) suggested that there was a greater spillover of H_2_ from fermentation in sheep fed twice a day than 8 times a day. Elevated H_2_ associated to rapid fermentation may also been a consequence of methanogens being inhibited by the low ruminal pH associated with the consumption of high concentrate diets (Janssen, 2010).

#### Interaction with pH

Greater increase in *[2H]_Pr_* was obtained by inhibiting methanogenesis at lower pH in batch, but not in continuous culture (not shown). Even though the range in pH was greater in continuous than in batch cultures, the mean (Table 1) and median final pH of batch cultures were numerically lower than pH of continuous cultures. Perhaps better buffered continuous cultures precluded detecting interactions between methanogenesis inhibition and pH. With the exception of the Slyter (1979) experiment in continuous culture in which the pH of the buffer was varied in some treatments, for the rest of the treatment means in the batch and continuous cultures databases, pH was a response to treatments, rather than a treatment itself. Controlled experiments of methanogenesis inhibition at different pH would have more power to detect the interaction between methanogenesis inhibition and pH on [H] sinks.

#### Type of CH_4_ production antagonist

Perhaps the most interesting result related to variation in [H] sinks among experiments was the lack of detection of H_2_ accumulation with strong methanogenesis inhibition with linoleic and linolenic acids (O'Brien et al., 2013). Other *in vitro* batch (van Nevel and Demeyer, 1981) and continuous (Czerkawski and Clapperton, 1984; Machmüller et al., 1998) culture (Table S4), and *in vivo* (Clapperton, 1974; Czerkawski and Clapperton, 1984) experiments with linseed oil, which is rich in linolenic acid, also found minimal or no H_2_ accumulation. On the other hand, Marty and Demeyer (1973) reported considerable H_2_ accumulation when inhibiting CH_4_ production in batch cultures with linseed oil. Czerkawski and Clapperton (1984) indicated that linseed oil stimulated microbial growth, which would be an alternative [H] sink to CH_4_ and H_2_, although the opposite result was reported by van Nevel et al. (1969).

It is interesting that linoleic and linolenic acid and linseed oil were strongly propionogenic with mixed (O'Brien et al., 2013) or chemically defined (van Nevel and Demeyer, 1981), but not with roughage (Fievez et al., 2007; O'Brien et al., 2013), substrates (Table S4). Indeed, between about 25 and 50% of [H] spared from methanogenesis was redirected toward propionate production with mixed (O'Brien et al., 2013) and chemically defined substrates (van Nevel and Demeyer, 1981) when methanogenesis was inhibited with linoleic or linolenic acids or linseed oil, which is numerically much higher than [H] redirection to propionate with most of the rest of the inhibitors (Table S4). In continuous culture, linseed supplementation stimulated propionate production disregarding the level of concentrate inclusion (Machmüller et al., 1998). Clapperton (1974) and Yang et al. (2009) reported increases in ruminal propionate concentration *in vivo* with linseed supplementation of mixed diets. Stimulation of propionate production as an alternative [H] sink could therefore partially explain lack of or small H_2_ accumulation when methanogenesis was inhibited using linoleic and linolenic acid with mixed and chemically defined substrates, although this explanation seems less likely with roughages. Other unsaturated oils such as some fish (Fievez et al., 2003) and algae (Fievez et al., 2007) oils in batch culture, and sunflower seed (Machmüller et al., 1998) and monolaurin (Klevenhusen et al., 2009) in continuous culture, were also associated with small H_2_ production and were propionogenic.

Research is needed to understand how linolenic acid and linseed oil promoted propionate production, and how that seems to be substrate-dependent. Linseed oil supplementation increased propionate concentration in the rumen, but succinate-producing *F. succinogenes* and *R. flavefaciens* numbers were decreased (Yang et al., 2009). In a batch culture experiment, both linoleic and linolenic acid stimulated propionate production and inhibited *F. succinogenes*, but effects on *R. flavefaciens* were less clear (Zhang et al., 2008). These results are in agreement with pure culture results in which both linoleic and linolenic acid were inhibitory to *F. succinogenes* and *R. flavefaciens*; however, other succinate and propionate producers such as *R. amylophilus, Prevotella* spp., *Mitsuokella multiacidus, S. ruminantium, Veilonella parvula*, and *Anaerovibrio lipolytica* were insensitive to linoleic and linolenic acids at the concentration tested (Maia et al., 2007). Greater toxicity of linoleic and linolenic acids to cellulolytic bacteria and fungi than to other species (Maia et al., 2007), might then explain why linoleic and linolenic acid stimulated propionate more with a mixed than with a roughage substrate (O'Brien et al., 2013). The response of non-cellulolytic succinate and propionate producers in mixed cultures to linoleic and linolenic acid, and linseed oil, would need to be studied.

Unsaturated fatty acids may also decrease H_2_ accumulation through biohydrogenation. It is true that the decrease of CH_4_ production through competition for [H] by fatty acids biohydrogenation is considered to be small (Nagaraja et al., 1997). However, because H_2_ accumulation when methanogenesis is inhibited is smaller than CH_4_ production in the typical, methanogenesis-uninhibited ruminal fermentation, the relative biohydrogenation effect on H_2_ accumulation would be more important than on CH_4_. Furthermore, the effect of biohydrogenation on H_2_ would be stoichiometrically greater than on CH_4_ by a 4 to 1 ratio. For example, linolenic acid at 1.25 ml/l decreased CH_4_ production by 60 and 70% with a mixed and a roughage substrate, respectively (O'Brien et al., 2013). Complete biohydrogenation of linolenic acid at 1.25 ml/l to stearic acid would have incorporated 14 and 18% of *[2H]_CH4_* in the control treatment (calculations not shown). This is greater than the average percentage of [H] diverted from CH_4_ to H_2_ in batch cultures at predicted 100% methanogenesis inhibition (calculation from Table 3), and thus, theoretically, it could explain lack of H_2_ accumulation. It should be considered, however, that 1.25 ml/l of linolenic acid represented about 11% of the substrate incubated and resulted in decreased DMD (O'Brien et al., 2013); less *[2H]_produced_*, would then also contribute to explain lack of detection of H_2_.

Unsaturated fatty acids have been shown to inhibit lipolysis and biohydrogenation (Noble et al., 1974; Beam et al., 2000; Boeckaert et al., 2007, 2008; Toral et al., 2012), but absolute [H] incorporated of into fatty acids double bonds might increase with oil supplementation, even if the proportion of reduced double bonds relative to the total double bonds available in fatty acids was decreased. The net result would depend on the balance between the limiting [H]-incorporating substrate (fatty acids double bonds) and the effect of the oil supplement on enzyme activity (inhibition of lipases, isomerases and reductases).

The classification of methanogenesis inhibitors into chemical inhibitors, ionophores, oils, and nitrate/nitrocompounds, was inaccurate to describe the effects of the different inhibitors on fermentation shifts. For example, two fish oils differed in the extent they caused H_2_ accumulation and stimulated propionate production when inhibiting methanogenesis (Fievez et al., 2003). It has been known that fatty acids differ on their effects on CH_4_ production, with polyunsaturated and medium-chain free fatty acids exerting the greatest inhibitory effects on methanogenesis (Beauchemin et al., 2008; Martin et al., 2010); from this analysis, it appears that fatty acids also differ regarding the consequences on [H] incorporation into other pathways when methanogenesis is inhibited. Also, a hexadecatrienoic acid from algae origin (Ungerfeld et al., 2005) caused considerable more H_2_ accumulation than a commercial algae product (Fievez et al., 2007). Content of algal secondary compounds such as isoprenoids and small halogenated compounds varies among algae lineages and is associated with the extent of methanogenesis inhibition algae exert *in vitro* (Machado et al., 2014); the type and content of secondary compounds can contribute to explain differences among algae and algae-based products on their effects on shifts in [H] sinks when methanogenesis is inhibited.

Nitrate caused somewhat less H_2_ accumulation than did nitrocompounds (not shown). Reduction of NO^−^_3_ to NH^+^_4_ is both thermodynamically and stoichiometrically very favorable (Thauer et al., 1977). Nitrate reduction would then outcompete H_2_ formation for [H], likely explaining the relatively small effect of NO^−^_3_ on H_2_ accumulation; however, *in situ* reduction of the nitro moiety in nitrocompounds (Anderson et al., 1993) could be thought to act similarly, and therefore nitrocompounds would be expected to cause equally small H_2_ accumulation as NO^−^_3_.

Inhibiting methanogenesis decreased *FH* in batch cultures. In contrast to this overall response, nitrocompounds increased *FH* in the experiment by Anderson et al. (2010). In other *in vitro* and *in vivo* experiments with nitrocompounds, total VFA concentration was generally not decreased (Anderson et al., 2003, 2006; Brown et al., 2011; Martínez-Fernández et al., 2014), perhaps because nitrocompounds might be partly metabolized to VFA. Nitrocompounds such as 3-nitropropionate may perhaps stimulate propionate production because they were partly converted to propionate. However, 3-carbon units 2-nitropropanol decreased propionate production, and on the other hand 2-carbon unit compounds nitroethane and nitroethanol increased propionate production (Anderson et al., 2003, 2010).

Martínez-Fernández et al. (2014) reported no effects of nitrocompounds on *in situ* apparent digestibility. Inhibiting methanogenesis *in vivo* using 3-nitrooxypropanol resulted in a quadratic tendency to increase dry matter and organic matter digestibility at the highest dose (Romero-Perez et al., 2014). A complete understanding of the effects of nitrocompounds on [H] sinks in the rumen is of much interest to applied animal production.

### Implications to animal production

In general, the main objective of increasing the output of heat of combustion in VFA through methanogenesis inhibition was little or not achieved. This was not because of the energetic inefficiency represented by H_2_ accumulation, as gaseous energy losses steadily decreased with the decline in CH_4_ production (Figure 7), but because of the inhibition of fermentation that generally accompanied methanogenesis inhibition. However, there was much variation among experiments and between *in vitro* systems. Methanogenesis inhibition using linoleic and linolenic acid was associated with little or no H_2_ accumulation, and no decrease in DMD in case of linoleic acid (although *FH* decreased). Also, nitrocompounds were not associated with a decrease in *FH*. As a result, nitrocompounds, and linoleic and linolenic acids at some concentrations, increased the output of heat of combustion in VFA.

It is generally accepted that elevated H_2_ pressure can hinder cofactors re-oxidation and thus inhibit fermentation (Wolin et al., 1997). In the present meta-analysis, H_2_ pressure negatively associated with *FH* and neutral detergent fiber digestibility, but not with DMD (not shown). Broudiscou et al. (2014) reported that the effect of headspace H_2_ pressure on *FH* in batch cultures was dependent on the source of inoculum. Some *in vivo* studies (Clapperton and Czerkawski, 1971; Cole and McCroskey, 1975; Reynolds et al., 2014) reported negative effects of methanogenesis inhibition on apparent digestibility, although most (Clapperton, 1974; Johnson, 1974; Cole and McCroskey, 1975; Kung et al., 2003; Knight et al., 2011; Mitsumori et al., 2012; Martínez-Fernández et al., 2014; Romero-Perez et al., 2014) did not, despite the elevated H_2_ reported in some of these experiments. Johnson (1972) reported a tendency for an interaction between methanogenesis inhibition and intake level on energy digestibility in sheep. Also working with sheep, Sawyer et al. (1974) reported an increase in digestibility of most feed fractions when CH_4_ production was inhibited. Neither apparent digestibility nor *FH* consider carbon from substrate digested and incorporated into microbial biomass; if methanogenesis inhibition stimulated microbial biomass production, it may decrease apparent but not true dry matter digestibility in some experiments. Effects of methanogenesis inhibition on true digestibility, and partition of digested and fermented substrate into fermentation products and microbial biomass need to be further studied in order to better understand the relationship between H_2_ pressure and fermentation and digestion.

If, as previously discussed, microbial biomass can be one alternative [H] sink to CH_4_, the additional microbial protein could benefit those animals not covering their metabolizable protein requirements. Some benefits in N retention in sheep when inhibiting CH_4_ production have been reported (Singh and Trei, 1972; Johnson, 1974). A recent experiment did not find effects of inhibiting methanogenesis on N excreted in urine, although the decrease in CH_4_ production was relatively small (Reynolds et al., 2014). General effects of methanogenesis inhibition on microbial anabolism and whole animal N metabolism are presently poorly understood, and research is needed to address this problem. It is important that methanogenesis inhibition experiments report formate as a [H] sink, as well as the more reduced VFA valerate and caproate and atypical reduced metabolites such as alcohols. In continuous cultures and *in vivo*, it is also possible that part of [H] when methanogenesis is inhibited was diverted to reductive acetogenesis, where existing reductive acetogens might have a greater opportunity to increase their numbers and/or reductive acetogenic activity over time under elevated H_2_ pressure. In agreement, in the present meta-analysis, numerically less H_2_ accumulated relative to methanogenesis inhibition in continuous than in batch cultures. It would be interesting if reductive acetogenesis could be stimulated *in vivo* when methanogenesis is inhibited.

With regard to the environmental impact of H_2_ emissions in the methanogenesis-inhibited rumen, H_2_ has a global warming potential of 5.8 compared to 25 of CH_4_ on a CO_2_-mass equivalent basis (Rooke et al., 2014). At predicted 100% methanogenesis inhibition, global warming potential of H_2_ was 9.8 and 5.8% of global warming potential of control treatments in batch and continuous cultures, respectively (not shown). Therefore, if the *in vitro* conditions analyzed applied *in vivo*, inhibiting methanogenesis would have been environmentally beneficial in spite of H_2_ accumulation. From a production point of view however, enthalpy in nutritionally useful fermentation products would have been decreased in many cases.

Batch and continuous cultures differed with regard to re-direction of [H] toward propionate and H_2_ when methanogenesis was inhibited. The implications of this analysis to the live animal could be quite different depending on which of the *in vitro* systems, if any, better reproduce the *in vivo* situation. Lack of *in vivo* data allowing calculation of complete [H] balances makes it difficult to conclude on whether batch or continuous cultures, or neither of the two, are a better model of the animal with regard to [H] shifts when ruminal methanogenesis is inhibited. This highlights the need for *in vivo* experiments on methanogenesis inhibition where VFA and gases production are simultaneously measured, studying also changes in true digestibility, microbial biomass production and chemical composition, and whole animal N metabolism, and the microbial community structure.

## Conclusions

The main findings of this meta-analysis are:

- Inhibiting methanogenesis resulted in a moderate increase in [H] incorporation into propionate production in batch cultures, and no increase in continuous cultures. There was no increase in butyrate in either system;
- Benefit of inhibiting methanogenesis in total energy output in VFA depended on the inhibitor and concentration used and on the *in vitro* system;
- Both in batch and continuous cultures, inhibiting methanogenesis resulted in a consistent decrease in the percentage of *[H]_produced_* recovered as the sum of [H] incorporated into propionate, butyrate, CH_4_ and H_2_.

## Conflict of interest statement

The author declares that the research was conducted in the absence of any commercial or financial relationships that could be construed as a potential conflict of interest.

## Abbreviations

Δ*H_gases_*: heat of combustion in methane and dihydrogen
Δ*H_VFA_*: heat of combustion in total volatile fatty acids
DMD: apparent DM digestibility
*FH* =: hexose-equivalent estimated fermented organic matter
[H]: metabolic hydrogen
[2H]_But_: metabolic hydrogen incorporated into butyrate production
[2H]_CH4_: metabolic hydrogen incorporated into CH_4_ production
[2H]_H2_: metabolic hydrogen incorporated into H_2_
[2H]_incorporated_: total incorporation of reducing equivalent pairs into propionate, butyrate, CH_4_ and H_2_
[2H]_produced_: total production of reducing equivalent pairs
[2H]_Pr_: metabolic hydrogen incorporated into propionate production
[2H]_recovery_: percentage of metabolic hydrogen produced incorporated into methane, propionate, butyrate and dihydrogen
VFA: volatile fatty acids.
