# Corrigendum: Shifts in metabolic hydrogen sinks in the methanogenesis-inhibited ruminal fermentation: a meta-analysis

**DOI:** 10.3389/fmicb.2015.00538

**Published:** 2015-06-02

**Authors:** Emilio M. Ungerfeld

**Affiliations:** Instituto de Investigaciones Agropecuarias (INIA) CarillancaTemuco, Chile

**Keywords:** methanogenesis inhibition, Rumen, fermentation, metabolic hydrogen, meta-analysis, volatile fatty acids

I wish to point out a mistake I made in the Discussion section of Ungerfeld, E. M. (2015) Shifts in metabolic hydrogen sinks in the methanogenesis-inhibited ruminal fermentation: a meta-analysis. Front. Microbiol. 6:37. doi: 10.3389/fmicb.2015.00037, which was kindly pointed out to me by Dr. Stéphane Duval. When citing the articles by Martínez-Fernández et al. (2014) and Romero-Perez et al. (2014), I mistakenly confused nitrooxycompounds with nitrocompounds. Both Martínez-Fernández et al. (2014) and Romero-Perez et al. (2014) evaluated nitrooxycompounds, not nitrocompounds, in their work. I wish to acknowledge this mistake and thank Dr. Stéphane Duval for contacting me on this.

## Conflict of interest statement

The author declares that the research was conducted in the absence of any commercial or financial relationships that could be construed as a potential conflict of interest.
