# Supplementary material for: Shifts in metabolic hydrogen sinks in the methanogenesis-inhibited ruminal fermentation: a meta-analysis
Source: Front Microbiol. 2015 Feb 4;6:37. doi: 10.3389/fmicb.2015.00037 (PMC4316778; doi:10.3389/fmicb.2015.00037)
Supplement: Supplementary file 1 [file Table1.DOCX]

Table S1. Studies used in the ruminal batch cultures meta-analysis on the effects of methanogenesis inhibition on metabolic hydrogen sinks.

| Study | Number of experiments | Number of treatment means | Substrate and amount (mg) | Volume (ml) | Inoculum species | Incubation (h) | CH_4_ production antagonists (%maximum decrease in CH_4_ production) |
| --- | --- | --- | --- | --- | --- | --- | --- |
| van Nevel *et al.* (1969) | 1 | 8 | pyruvic acid, 38 | 30 | ovine | 4 or 15 | chloral hydrate (100) |
| Chalupa *et al.* (1980) | 4 | 35 | mixed or high-concentrate, 500 | 40 | ovine | 16 | monensin (41) and amicloral (100) |
| van Nevel and Demeyer (1981) | 2 | 10 | cellobiose and maltose (amounts not provided) | 50 | ovine | 2 | linseed oil hydrolysate (94) and chloral hydrate (100) |
| Nollet *et al.* (1997) | 1 | 3 | roughage, 500 | 50 | ovine | 24 | 2-bromoethanesulphonic acid |
| Ungerfeld *et al.* (2003) | 1 | 4 | roughage, 300 | 50 | bovine | 24 | propynoic acid (76) |
| Fievez *et al.* (2003) | 1 | 16 | roughage, 400 | 20 | ovine | 48 | fish oils (78) and soybean oil (64) |
| Ungerfeld *et al.* (2005) | 1 | 7 | roughage, 250 | 50 | bovine | 24 | algal fatty acid (97) |
| Ungerfeld *et al.* (2006) | 4 | 12 | roughage, 400 or 500 | 50 | bovine | 24 | propynoic acid (69), ethyl-2-butynoate (100) |
| Fievez *et al.* (2007) | 3 | 18 | roughage, 400 | 20 | ovine | 24 | algae oil (87), sunflower oil (17), linseed oil (0) |
| Goel *et al.* (2009) | 1 | 4 | high-concentrate, 500 | 20 | bovine | 24 | capric acid (88) |
| Guo *et al.* (2009) | 1 | 3 | cellulose/starch, 200 | 40 | bovine | 24 | nitrate (74) |
| Anderson *et al.* (2010) | 1 | 7 | roughage, 200 | 10.1 | bovine | 24 | dimethyl-2-nitroglutarate (97),  2-nitromethylpropionate (98), nitroethane (99) |
| Lin *et al.* (2011) | 1 | 2^a^ | cellulose/starch, 200 | 50 | bovine | 24 | sodium nitrate (79) |
| O'Brien *et al.* (2013) | 6 | 64 | roughage or mixed | 50 | bovine | 24 | lauric (99), linoleic (83) and linolenic acid (100), 2-bromoethanesulphonic acid (57), bromochloromethane (100), pyromellitic diimide (100) |

^a^Treaments with centrifuged ruminal fluid as inoculum excluded.
