# Supplementary material for: Shifts in metabolic hydrogen sinks in the methanogenesis-inhibited ruminal fermentation: a meta-analysis
Source: Front Microbiol. 2015 Feb 4;6:37. doi: 10.3389/fmicb.2015.00037 (PMC4316778; doi:10.3389/fmicb.2015.00037)
Supplement: Supplementary file 2 [file Table2.DOCX]

Table S2. Studies used in the ruminal continuous cultures meta-analysis on the effects of methanogenesis inhibition on metabolic hydrogen sinks.^a^

| Study | Number of experiments | Number of treatment means | Substrate and amount (g DM/d) | Liquid dilution rate (h^-1^) | CH_4_ production antagonists (%maximum decrease in CH_4_ production) |
| --- | --- | --- | --- | --- | --- |
| Slyter and Wolin (1967) | 1 | 4 | high concentrate, 17.4 | 0.0625 | copper sulphate (92) |
| Slyter (1979) | 1 | 6 | roughage, 12.1 | 0.0625 | monensin (33), dichloroacetamide (100), dichloroacetamide + monensin (100), low pH (96), low pH + monensin (98) |
| Stanier and Davies (1981) | 3 | 9 | mixed, 16.7 | 0.0188 to 0.0417 | 2-trichloromethyl-4-dichloromethylene benzo[1,3] dioxin-6-carboxylic acid (87), monensin (53) |
| García-López *et al.* (1996) | 1 | 2 | high concentrate, 18 | 0.0667 | 9, 10-anthraquinone (62) |
| Dong *et al.* (1997) | 1 | 8 | roughage or high concentrate, 10 | 0.025 | canola oil (44), cod liver oil (57), coconut oil (88) |
| Machmüller *et al.* (1998) | 1 | 6 | mixed or high concentrate, 11 or 22 | 0.0233 | ruminally protected fat (10), coconut oil (50), rapeseed (14), sunflower seed (31), linseed (27) |
| Dohme *et al.* (1999) | 1 | 4 | mixed, 11.1 | 0.0242 | coconut oil (79), defaunation (66) |
| Machmüller *et al.* (2001) | 2 | 8 | roughage or high-concentrate, 11.2 to 15.0 | 0.0231 | coconut oil (62), lauric acid (82) |
| Machmüller *et al.* (2002) | 1 | 4 | mixed, 13.0 to 13.8 | 0.025 | lauric acid (78) |
| Soliva *et al.* (2004) | 1 | 8 | mixed, 14 | 0.0208 | lauric and myristic acid combinations (71) |
| Klevenhusen *et al.* (2009) | 1 | 8 | roughage or mixed, 15 | 0.0165 | monolaurin (53) |
| Watanabe *et al.* (2010) | 1 | 4 | high concentrate, 12 | 0.0208 | cashew nut shell liquid (70) |
| Soliva *et al.* (2011) | 1 | 8 | mixed, 40 | 0.0196 | garlic oil (91), allyl isothiocyanate (39), lovastatin (38), chenodeoxycholic acid (0.9), 3-azido-propionic acid ethyl ester (98), levulinic acid (6.3), 4-[(pyridine-2-ylmethyl)-amino]-benzoic acid (-56) |

^a^Bovine ruminal contents were used as inoculum in all experiments in continuous cultures.
