# Supplementary material for: Shifts in metabolic hydrogen sinks in the methanogenesis-inhibited ruminal fermentation: a meta-analysis
Source: Front Microbiol. 2015 Feb 4;6:37. doi: 10.3389/fmicb.2015.00037 (PMC4316778; doi:10.3389/fmicb.2015.00037)
Supplement: Supplementary file 3 [file Table3.DOCX]

Table S3. Effect of type of substrate on shift of metabolic hydrogen towards propionate, butyrate and dihydrogen.

| *In vitro* system | Inhibitors | Substrate | *Δ[2H]_Pr_/ Δ[2H]_CH4_* (mol/mol) | *Δ[2H]_But_/ Δ[2H]_CH4_* (mol/mol) | *Δ[2H]_H2_/ Δ[2H]_CH4_* (mol/mol) | References |
| --- | --- | --- | --- | --- | --- | --- |
| Batch cultures | lauric acid, linoleic acid, linolenic acid, bromochloromethane,  2-bromoethanesulfonate, pyromellitic diimide | roughage | -0.012 ± 0.024  (*p* = 0.62) | 0.068 ± 0.010 (*p* < 0.001) | -0.073 ± 0.014  (*p* < 0.001) | O'Brien *et al.* (2013) |
|  |  | mixed | -0.23 ± 0.030  (*p* < 0.001) | 0.044 ± 0.012 (*p* = 0.001) | -0.099 ± 0.029  (*p* = 0.003) |  |
| Continuous cultures | coconut oil, canola oil, cod liver oil | roughage | 1.40 ± 0.97  (*p* = 0.28) | 0.059 ± 0.11  (*p* = 0.64) | -0.0055 ± 2.7 × 10^-4^  (*p* = 0.002) | Dong *et al.* (1997) |
|  |  | high concentrate | -0.35 ± 0.17  (*p* = 0.18) | 0.29 ± 0.080  (*p* = 0.07) | -0.012 ± 0.0096  (*p* = 0.33) |  |
|  | coconut oil, lauric acid | mixed | 0.34 ± 0.53  (*p* = 0.58) | -0.13 ± 0.18  (*p* = 0.54) | -0.055 ± 0.055  (*p* = 0.42) | Machmüller *et al.* (2001) |
|  |  | high concentrate | 0.15 ± 0.49  (*p* = 0.79) | 0.018 ± 0.059 (*p* = 0.79) | -0.082 ± 0.023  (*p* = 0.07) |  |
|  | monolaurin | roughage | -0.15 ± 0.11  (*p* = 0.25) | 0.19 ± 0.17  (*p* = 0.35) | -0.0021 ± 0.0032  (*p* = 0.56) | Klevenhusen *et al.* (2009) |
|  |  | mixed | -0.65 ± 8.8 × 10^-4^  (*p* < 0.001) | 0.61 ± 0.26  (*p* = 0.26) | -0.020 ± 0.0047  (*p* = 0.15) |  |
