# Supplementary material for: Shifts in metabolic hydrogen sinks in the methanogenesis-inhibited ruminal fermentation: a meta-analysis
Source: Front Microbiol. 2015 Feb 4;6:37. doi: 10.3389/fmicb.2015.00037 (PMC4316778; doi:10.3389/fmicb.2015.00037)
Supplement: Supplementary file 4 [file Table4.DOCX]

Table S4. Effect of type of methanogenesis inhibitor on shift of metabolic hydrogen towards propionate, butyrate and dihydrogen.

| Type of inhibitor | Compounds /additives /ingredients | *In vitro* system | *Δ[2H]_Pr_/ Δ[2H]_CH4_* (mol/mol) | *Δ[2H]_But_/ Δ[2H]_CH4_* (mol/mol) | *Δ[2H]_H2_/ Δ[2H]_CH4_* (mol/mol) | References |
| --- | --- | --- | --- | --- | --- | --- |
| Chemicals directly inhibiting methanogenesis | amicloral,  chloral hydrate, bromochloromethane,  2-bromoethanesulfonate, ethyl-2-butynoate, propynoic acid, pyromellitic diimide | batch cultures | -0.094 ± 0.012 (*p* < 0.001) | -0.031 ± 0.0098 (*p* = 0.002) | -0.21 ± 0.018 (*p* < 0.001) | van Nevel *et al.* (1969); Chalupa *et al.* (1980); van Nevel and Demeyer (1981); Nollet *et al.* (1997); Ungerfeld *et al.* (2003, 2006); O'Brien *et al.* (2013) |
| Ionophores | monensin | batch cultures | -0.46 ± 0.076 (*p* = 0.004) | 0.20 ± 0.057  (*p* = 0.023) | -0.021 ± 0.0015  (*p* < 0.001) | Chalupa *et al.* (1980) |
| Nitrate /nitrocompounds | NO_3_^-^, dimethyl-2-nitroglutarate, nitroethane, 2-nitro-methyl-propionate | batch cultures | -0.018 ± 0.080 (*p* = 0.83) | 0.17 ± 0.061  (*p* = 0.032) | -0.0080 ± 0.0035  (*p* = 0.06) | Guo *et al.* (2009); Anderson *et al.* (2010); Lin *et al.* (2011) |
| Oils (all) | algae oil, fish oil, linseed oil hydrolysate, lauric acid, linoleic acid, linolenic acid | batch cultures | -0.22 ± 0.59  (*p* = 0.40) | 0.12 ± 0.055  (*p* = 0.039) | -0.024 ± 0.0084  (*p* = 0.007) | van Nevel and Demeyer (1981); Fievez *et al.* (2003); Ungerfeld *et al.* (2005); Fievez *et al.* (2007); Goel *et al.* (2009); O'Brien *et al.* (2013) |
| Oils (linoleic and linolenic acid with mixed and chemically defined substrates) | linoleic and linolenic acid and linseed oil hydrolysate | batch cultures | -0.43 ± 0.067 (*p* < 0.001) | 0.15 ± 0.023  (*p* < 0.001) | 3.2 × 10^-4^ ± 8.0 × 10^-4^  (*p* = 0.70) | van Nevel and Demeyer (1981); O'Brien *et al.* (2013) |
| Oils (linoleic and linolenic acid with roughage substrates | linoleic and linolenic acid | batch cultures | -0.046 ± 0.038 (*p* = 0.27) | 0.15 ± 0.013  (*p* < 0.001) | 0 ± 0^c^ | O'Brien *et al.* (2013) |
| Chemicals directly inhibiting methanogenesis | anthraquinone, allyl isothiocyanate, lovastatin, chenodexyocholic acid, 3-azido-propionic acid ethyl ester, levulinic acid, APEE^a^, ICI 111075^b^, dicloroacetamide | continuous cultures | 0.18 ± 0.12  (*p* = 0.18) | -0.29 ± 0.070  (*p* = 0.009) | -0.18 ± 0.019 (*p* < 0.001) | Slyter (1979); Stanier and Davies (1981); García-López *et al.* (1996); Soliva *et al.* (2011) |
| Ionophores | monensin | continuous cultures | -0.34 ± 0.044 (*p* = 0.005) | 0.037 ± 0.051  (*p* = 0.52) | 3.9 × 10^-6^ ± 3.0 × 10^-4^  (*p* = 0.99) | Slyter (1979); Stanier and Davies (1981) |
| Oils (all) | canola oil, canola seed, sunflower seed, linseed, coconut oil, cod liver oil, garlic oil, lauric acid, myristic acid, monolaurin | continuous cultures | -0.13 ± 0.073 (*p* = 0.089) | 0.17 ± 0.065  (*p* = 0.016) | -0.048  (*p* < 0.001) | Dong *et al.* (1997); Machmüller *et al.* (1998); Dohme *et al.* (1999); Machmüller *et al.* (2001); Machmüller *et al.* (2002); Soliva *et al.* (2004); Klevenhusen *et al.* (2009); Soliva *et al.* (2011) |
| Oils (linseed) | linseed | continuous cultures | -0.30^d^ | 0.74^d^ | 0.014^d^ | Machmüller *et al.* (1998) |
| Antiprotozoal | copper sulfate | continuous cultures | 0.39 ± 0.70  (*p* = 0.63) | 0.082 ± 0.18  (*p* = 0.69) | -0.29 ± 0.35 (*p* = 0.49) | Slyter and Wolin (1967) |
| Plant extract | cashew nut shell liquid | continuous cultures | -1.35 ± 0.27  (*p* = 0.037) | 0.19 ± 0.19  (*p* = 0.41) | -0.025 ± 0.014  (*p* = 0.22) | Watanabe *et al.* (2010) |

^a^4-[(pyridin-2-methyl)-amino]-benzoic acid.

^b^2-trichloromethyl-4-dichloromethylene benzo[ 1,3] dioxind-carboxylic acid.

^c^H_2_ was not detected.

^d^Slope calculated with only two treatment means precluded calculating standard errors and *p* values.
